# Genome-Wide Comprehensive Analysis of the *GASA* Gene Family in *Populus*

**DOI:** 10.3390/ijms222212336

**Published:** 2021-11-15

**Authors:** Shuo Han, Zhiyin Jiao, Meng-Xue Niu, Xiao Yu, Mengbo Huang, Chao Liu, Hou-Ling Wang, Yangyan Zhou, Wei Mao, Xiaofei Wang, Weilun Yin, Xinli Xia

**Affiliations:** 1 National Engineering Laboratory for Tree Breeding, College of Biological Sciences and Technology, Beijing Forestry University, Beijing 100083, China; hanhan@bjfu.edu.cn (S.H.); jiaozy@bjfu.edu.cn (Z.J.); niumengxue@bjfu.edu.cn (M.-X.N.); yuxiao@bjfu.edu.cn (X.Y.); hmb425069@bjfu.edu.cn (M.H.); liuchao1306@bjfu.edu.cn (C.L.); whling@bjfu.edu.cn (H.-L.W.); 2 Salver Academy of Botany, Rizhao 276800, China; zhou@salver.cn (Y.Z.); mao@salong-agriculture.com (W.M.); wang@dfy.net.cn (X.W.)

**Keywords:** *GASA* genes, bioinformatics, *Populus*, growth and development, phytohormone

## Abstract

Gibberellic acid-stimulated *Arabidopsis* (GASA) proteins, as cysteine-rich peptides (CRPs), play roles in development and reproduction and biotic and abiotic stresses. Although the *GASA* gene family has been identified in plants, the knowledge about GASAs in *Populus euphratica*, the woody model plant for studying abiotic stress, remains limited. Here, we referenced the well-sequenced *Populus trichocarpa* genome, and identified the GASAs in the whole genome of *P. euphratica* and *P. trichocarpa*. 21 candidate genes in *P. trichocarpa* and 19 candidate genes in *P. euphratica* were identified and categorized into three subfamilies by phylogenetic analysis. Most GASAs with signal peptides were located extracellularly. The *GASA* genes in *Populus* have experienced multiple gene duplication events, especially in the subfamily A. The evolution of the subfamily A, with the largest number of members, can be attributed to whole-genome duplication (WGD) and tandem duplication (TD). Collinearity analysis showed that WGD genes played a leading role in the evolution of *GASA* genes subfamily B. The expression patterns of *P. trichocarpa* and *P. euphratica* were investigated using the PlantGenIE database and the real-time quantitative PCR (qRT-PCR), respectively. *GASA* genes in *P. trichocarpa* and *P. euphratica* were mainly expressed in young tissues and organs, and almost rarely expressed in mature leaves. *GASA* genes in *P. euphratica* leaves were also widely involved in hormone responses and drought stress responses. GUS activity assay showed that PeuGASA15 was widely present in various organs of the plant, especially in vascular bundles, and was induced by auxin and inhibited by mannitol dramatically. In summary, this present study provides a theoretical foundation for further research on the function of *GASA* genes in *P. euphratica*.

## 1. Introduction

Cysteine-rich peptides (CRPs), with a secretory peptide signal at the N-terminal region and a cysteine-rich domain containing 4–16 cysteine residues at the C-terminal region, are generally small (usually less than 160 amino acid residues) and have been increasingly studied in plant [1,2,3,4]. CRPs, as plant peptide ligands that trigger membrane receptors, play a vital role in plant development and reproduction, plant-bacteria symbiosis, and plant defense, and are widely recognized as important signaling components that coordinate cell-cell communication in plants [1,5,6,7]. Gibberellic acid-stimulated *Arabidopsis* (GASA) proteins are classified as cysteine-rich peptides consisting of a signal peptide at the N-terminal, a highly variable hydrophilic region in the middle, and a conserved region containing 12 cysteine residues at the C-terminal (GASA domain) [8]. GASA proteins are widely found in vascular terrestrial plants such as pteridophytes, gymnosperms, and angiosperms, which have significance for adapting to complex and diverse land life [9]. Since gibberellin-stimulated transcript 1 (GAST1) was first identified in tomato GA-deficient *gib1* mutant, an increasing number of GASA members have been identified over the past three decades [10].

Snakin-1 (SN1) and snakin-2 (SN2), which were classified as members of the GASA family, have antibacterial activity. Overexpression of *SN1* in potatoes and *SN2* in tomatoes inhibited the invasion of pathogenic bacteria and enhanced the tolerance to bacterial and fungal presence [11,12]. Likewise, the allogenic expression of *StSN1* could improve the tolerance to fungal pathogens in transgenic lettuce plants [13]. PdSN1, the homologous protein of potato snakin-1 in *Peltophorum dubium*, inhibited the growth of multiple plants and animal pathogens in vitro [14]. GASA proteins also participate in plant growth and development [15,16,17,18,19]. The silencing of *SN1* in potatoes affected cell division, primary metabolism and cell wall composition of transgenic plants, eventually leading to changes in leaf size and shape and plant dwarfing [20]. AtGASA5 delayed flowering time by enhancing the expression of *FLC* and repressing the expression of *FT* and *LEAFY* [17]. *GhGEG,* a gene from the *GASA* gene family in *Gerbera hybrida*, was directly transcriptional activated by *GhMIF* and *GhEIL1* to inhibit the ray petal elongation and cell elongation during petal development [21,22]. AtGASA4 regulated the differentiation and development of flower development meristem and increases the seed size and yield of *Arabidopsis*. [23]. FaGAST2 and FaGAST1 are involved in regulating the size of strawberry fruits, during the development and ripening of strawberry fruits [16]. *AtGASA10* is strongly expressed in developing anthers, seeds, and the vasculature of elongating roots, and overexpression of *AtGASA10* in transgenic *Arabidopsis* might reduce the length of the silique by affecting cell elongation [24]. And ZmGSL1, ZmGSL2, ZmGSL4, ZmGSL6, and ZmG*SL9*, GASAs in *Zea mays,* contributed to lateral root formation and development in maize [19,25].

GASAs are also involved in the transduction of hormone signals [26]. OsGSR1 in the rice *GASA* gene family, a positive regulator of GA signaling, interacted with DIM/DWF1 to regulate BR synthesis, and mediated the interaction between BR and GA signaling pathways [8]. As an integrator of gibberellin, abscisic acid and glucose signaling, *AtGASA6* connected the functions of *RGL2* and *AtEXPA1*, and regulated seed germination and hypocotyl length [18]. The expression of *GASA* family genes was responsive to exogenous treatment of growth hormones and stress hormones [15,21,27,28,29]. Exogenous GA_3_ inhibited the expression of most *MdGASA* genes in apple flowers, except the *AtGASA10* homologous gene *MdGASA1/6/7/19*, *HbGASA7-1*, *HbGASA14-3*, *HbGASA15* were induced by stress hormones salicylic acid, abscisic acid, ethylene and methyl jasmonate in *Hevea brasiliensis* [27]. The *GASA* genes participate in the regulation of plant strategies under stress. For example, transcriptomic and qRT-PCR analyses revealed the expression of *GASA*s was induced or inhibited by abiotic stresses such as cold, heat, drought, osmotic, salt, and wounding [25,30,31]. Overexpression of *AtGASA14* reduced the accumulation of reactive oxygen species (ROS) to resist ABA and high salt stress in transgenic *Arabidopsis* [32]. The allogenic expression of *SmGASA4* was found to promote the development of *Arabidopsis* roots and enhance plant resistance to salt and drought stress [31].

Plants are frequently subjected to various environmental stresses, especially abiotic stresses, which inhibit plant growth and biomass accumulation. *P. euphratica*, which can survive in extremely arid desert ecosystems, is widely regarded as an important model plant for studying woody plants in response to abiotic stresses [33,34,35,36]. In our previous studies, two *GASA* genes were abundant in transcripts, one of which was homologous to *AtGASA1* and significantly up-regulated in drought-stressed *P. euphratica* leaves [37,38]. However, *GASA1* was down-regulated by drought in *Arabidopsis* [39]. Although they are homologous genes from different plants, they have different expression patterns in response to drought stress. It is necessary to identify the *GASA* gene family and research the functions of the *GASA* gene family in the growth and development and stress response of *P. euphratica*. However, little information concerning *GASA* genes in *P. euphratica* has been known. Recently, the genome of *P. euphratica* has been sequenced and assembled, making it possible to study the *GASA* gene family on a genome-wide scale [36,40]. While the full genome of *P. euphratica* v1.0 had not yet been assembled into chromosomes, and there were numerous gaps in the scaffolds [40,41]. The GASA proteins we searched in *P. euphratica* v2.0 were less than *P. euphratica* v1.0.

The genome of *P. trichocarpa* (black cottonwood) was well-sequenced and annotated and had high similarity and collinearity with that of *P. euphratica*. To ensure the reliability of the results, the *GASA* gene family was identified in the genome-wide of *P. euphratica* by referring to the *GASA* gene family of *P. trichocarpa* [42]. In this study, we identified 21 *PtrGASA* genes and 19 *PeuGASA* candidate genes. Chromosomal localization, gene expansion, gene structure, and upstream promoter *cis*-acting elements of candidate *GASA* genes were predicted and analyzed. The chemical properties, subcellular localization, motifs, and phylogenetic relationships of the proteins encoded by them were also predicted and analyzed. Furthermore, the expression patterns of these genes in different organs and tissues and their responses to drought stress and exogenous hormones were investigated. The temporal and spatial expression patterns of the representative gene *PeuGASA15* and its response to hormones and stresses were analyzed. This study represents the first comprehensive analysis of *GASA* genes in *P. euphratica* and *P. trichocarpa* and provided a theoretical basis for further study on the structures and functions of *Populus GASA* genes. Our findings might serve as a valuable resource for future studies of *GASA* genes related to vascular tissue development in *P. euphratica* as well as in other woody plants.

## 2. Results

### 2.1. Genome-Wide Identification of GASA Genes in Populus

To identify the members of the *GASA* gene family in *P. euphratica*, we searched for the conserved GASA domain (PF02704) in the whole genome protein database reported by Ma et al. and Zhang et al. [36,40]. We found 19 candidate GASA protein sequences in the protein database reported by Ma et al., two of which were encoded by the same gene LOC105128593 in different splicing ways (Table 1a). Fourteen GASA protein sequences were found in the protein database reported by Zhang et al., and 12 of which were identical to those reported by Ma et al. (Table 1b). *GWHGAAYU001024* is homologous to *AtGASA14* and encodes 158 amino acids. However, the same gene LOC105141680 encodes 222 amino acids as reported by Ma et al., which may be due to different splicing patterns in the intermediate hydrophilic region. The protein sequence of GWHPAAYU001570 was 98.86% identical with the homologous protein sequence in *P. trichocarpa*. Combined with two protein databases, we identified 19 GASA proteins in *P. euphratica* and named them according to gene ID on NCBI (Supplementary Table S1). 21 *GASA* genes with conserved GASA domains were identified in *P. trichocarpa* on Popgenie and named according to their position on chromosomes (Supplementary Table S1).

**Figure 1:**
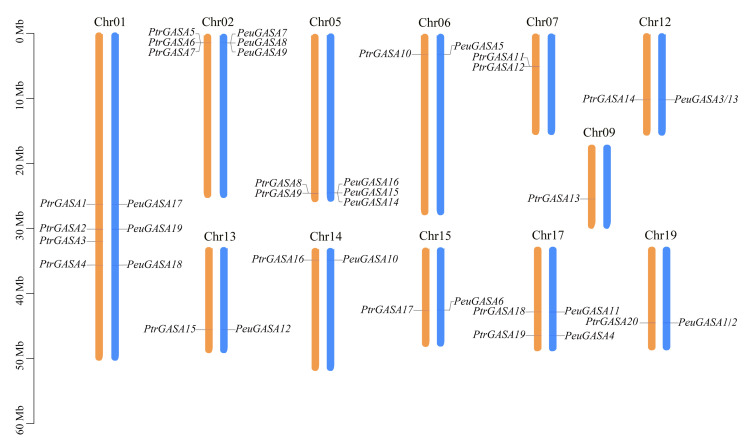
Positions of *PtrGASA* and *PeuGASA genes* on *P. trichocarpa* chromosomes. The *P. trichocarpa* chromosomes where the *PtrGASA* genes are located are shown in orange. The reference *P. trichocarpa* chromosomes where the *PeuGASA* genes are located are shown in blue. The positions of genes and the size of chromosomes can be estimated using the scale on the left of the picture.

Except for the fact that *PtrGASA21* was not assembled on any chromosome, *PtrGASA* genes were distributed on 12 chromosomes in *P. trichocarpa* genome (Figure 1). Four *PtrGASA* genes were found on chromosome 1, three *PtrGASA* genes were found on chromosomes 2, two *PtrGASA* genes were found on chromosomes 5, 7, and 17 respectively, and only one *PtrGASA* gene was found on the remaining seven chromosomes. According to the positions of homologous *PtrGASA*s or linked genes, the possible chromosomal position of *PeuGASA*s was determined (Supplementary Table S1 and Figure 1). Almost all *PeuGASA*s had a corresponding position on the *P. trichocarpa* chromosomes except for two pairs of *PeuGASA* genes (*PeuGASA1* and *PeuGASA2*; *PeuGASA3* and *PeuGASA13*). Three *PeuGASA* genes were found on chromosomes 1, 2, and 5 of *P. trichocarpa*, which were the chromosomes containing the most *PeuGASA* genes. *PtrGASA* duplicate gene clusters were found on chromosomes 2, 5, and 7 in *P. trichocarpa*, while no corresponding *PeuGASA* homologous gene or *PeuGASA* duplicate gene cluster was found on chromosome 7.

### 2.2. Evolutionary Relationships Analysis among GASA Domains and Collinearity Analysis among GASA Genes

**Figure 2:**
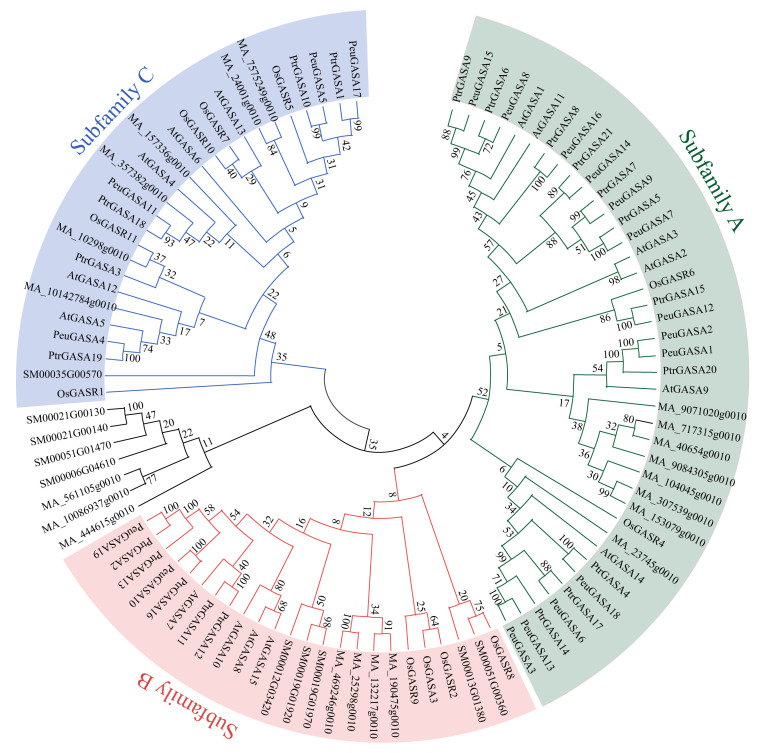
Neighbor-Joining phylogenetic tree of GASA domains. The 97 conserved GASA domains of GASAs in sequenced plants, *Selaginella moellendorffii*, *Picea abies*, *Oryza sativa* ssp. *japonica*, *Arabidopsis thaliana*, *Populus trichocarpa*, and *Populous euphratica* were analyzed. The tree was divided into three phylogenetic subgroups, which were shown in different colors.

To illustrate the evolutionary relationship of the *GASA* gene family, the phylogenetic tree of the conserved GASA domains was constructed. Except for the GASA domains of *S. moellendorffii* and *P. abies*, which were separated into a distal branch, the remaining GASA domains were divided into three subfamilies: subfamily A (green), subfamily B (red), subfamily C (blue) (Figure 2). Compared with the other two subfamilies, subfamily A had more members and higher rates of substitution. The GASA domain of *S. moellendorffii* was not found in the subfamily A. MA_9071020g0010, MA_40654g0010, MA_9084305g0010, MA_717315g0010, MA_104045g0010, MA_153079g0010, and MA_307539g0010, with low substitution rates, clustered together and diverged from PtrGASA20, PeuGASA1, PeuGASA2, and AtGASA9. While PtrGASA4, PeuGASA18, PtrGASA17, PeuGASA6, PtrGASA14, PeuGASA13, and PeuGASA3, with high substitution rates, clustered together and separated hierarchically from OsGASR4, MA 23745g0010, and AtGASA14 which evolved earlier. In subfamily B, the GASA domains of *S*. *moellendorffii*, *P. abies*, *O. sativa*, *A. thaliana*, and *Populus* each had a successive independent clade within the species. OsGASR8 and SM00051G00360, with similar sequences and high substitution rates, clustered together and diverged from SM00013G01380. OsGASR1 and SM00035G00570, with relatively low substitution rates, separated earlier in the subfamily C hierarchically. In the subfamily C, except for a pair of GASA domains of *P. abies* and the homologous GASA domains of *Populus*, the GASA domains dispersed on several small branches had low substitution rates. Compared with GASA domains of other species, GASA domains of *Arabidopsis* and *Populus* had higher substitution rates and homology.

**Figure 3:**
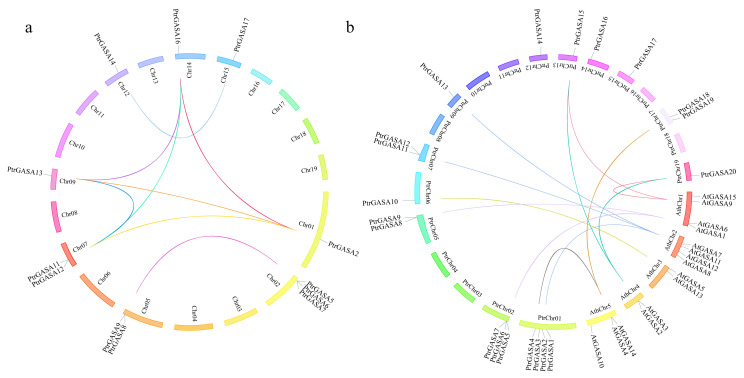
Analysis of the evolutionary relationship among *GASA* genes. (**a**) Evolution analysis of *GASA* gene family in *P. trichocarpa*; (**b**) Collinear analysis of *GASA* gene family between the *Arabidopsis* and *P. trichocarpa*. Different sizes of fan rings represented different sizes of chromosomes. The colored lines represented collinear *GASA* gene pairs.

To illustrate the expansion pattern of *GASA* genes, we analyzed the duplicate events in the genomes of *P. trichocarpa* and *P. euphratica* (Table 2). Multiple duplicate events occurred in the evolution of *PtrGASA*s and *PeuGASA*s. Each pair in *PtrGASA2*, *PtrGASA11*, *PtrGASA13*, and *PtrGASA16* was a WGD pair, indicating that they all share a common ancestor. The WGD pairs and TD pairs of the *PeuGASA*s and *PtrGASA*s homologous genes were almost identical. The lack of duplicate genes in *PtrGASA*s and *PeuGASA*s, which may be due to genes loss caused by the chromosome dynamic rearrangement after whole-genome duplications. The *Ka/Ks* values of each duplicated pair were much less than 1, indicating that these genes were selected for purification. The *Ka/Ks* values of the duplicated pairs in *P. euphratica* were slightly higher than those in *P. trichocarpa*, indicating that *GASA* genes in *P. trichocarpa* might be more conserved. Unlike the multiple pairs of duplications in poplar, only a WGD pair and a TD pair were found in *Arabidopsis* (*AtGASA3* and *AtGASA9*, *AtGASA2* and *AtGASA3*). Collinear relationships of *GASA*s in poplar and *Arabidopsis* showed that 21 pairs of homologous genes were found in *P. trichocarpa* and *Arabidopsis*, which were composed of 7 *AtGASA* genes and 15 *PtrGASA* genes. There were 12 pairs of homologous genes between *P. trichocarpa* and *Arabidopsis* (Figure 3).

### 2.3. Gene Structure and Motif Identification, Physicochemical Properties, and Subcellular Localization of GASA Gene Family

**Figure 4:**
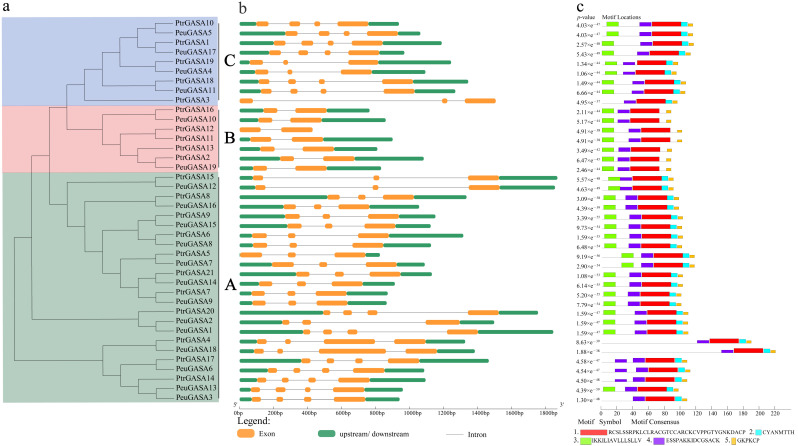
Gene structures and motif analysis of *GASA* gene family in *P. euphratica* and *P. trichocarpa*. (**a**) Phylogenetic tree of PtrGASAs and PeuGASAs. Subfamily A/B/C were represented by green, red and blue respectively; (**b**) Gene structures of *PtrGASA*s and *PeuGASA*s. Upstream or downstream non-coding regions were represented by green rounded rectangles, exons by orange rounded rectangles, and introns by gray lines; (**c**) Conserved motifs of PtrGASAs and PeuGASAs. Conservative motifs were presented in different colored boxes. The length of each nucleotide sequence or protein sequence can be estimated using the scale below the picture.

To illustrate the diversity of *GASA* genes in poplar, the arrangement of introns-exons was compared according to their phylogenetic relationships (Figure 4). As shown in Figure 4b, genes in subfamily B were composed of two exons and one intron. Except for *PtrGASA3*, *GASA* genes which were present in *P. trichocarpa* but had no corresponding homologous genes in *P. euphratica* belong to this subfamily. The members of the *GASA* gene family contain no more than three introns. The predicted motifs of GASA proteins were mainly concentrated at the C-terminal of GASA proteins. GASA domain was jointly constituted by motif 1, motif 2, motif 4, and motif 5. Motif 3, which was located at the N-terminal of the proteins and contained a large amount of the hydrophobic amino acid leucine. The multiple sequence alignment identity of 40 GASA proteins was only 26.70%, while that of 40 GASA domains was 66%. The conserved domain of GASA was composed of 59 or 60 amino acids, including 12 cysteine residues with conserved positions (Supplementary Figure S1a). Despite the lack of motif 2 and low similarity with other subfamily amino acid sequences, GASA proteins in subfamily B had conserved GASA domains.

**Figure 5:**
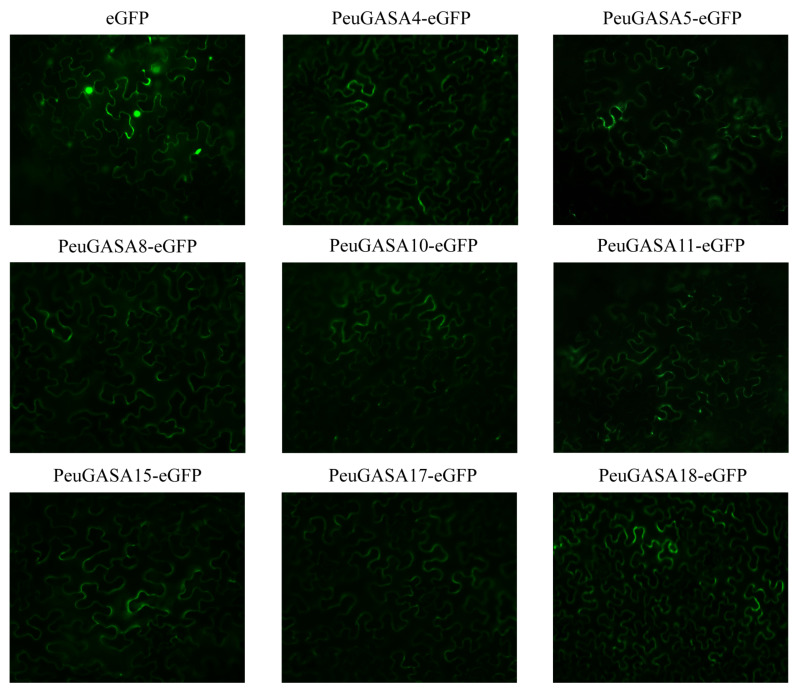
Subcellular localization of PeuGASA proteins. The Super::GFP and Super::PeuGASA-GFP fusion proteins were transiently expressed in tobacco.

*GASA* genes in *Populus* encoded 88–118 amino acids, except for *AtGASA14* homologous genes *PeuGASA18* and *PtrGASA4*, which encoded about 200 amino acids. The molecular weight of GASA proteins was relatively small, almost less than 13kDa, except for PeuGASA18 and PtrGASA4. The isoelectric point of GASA proteins was ranged from 7.96 to 9.76, which belonged to basic protein. The GASAs in *Populus* were mainly unstable proteins and signal peptides secreted through the classical secretion pathway (Supplementary Tables S2 and S3). The predicted subcellular localization of GASA proteins in *P. euphratica* and *P. trichocarpa* mainly in extra (Supplementary Table S2). Consistent with previously predicted subcellular localization, the GASA proteins in *P. euphratica* were localized in the extracellular region (Figure 5).

### 2.4. Expression Patterns of GASA Genes in Different Tissues of Populus

**Figure 6:**
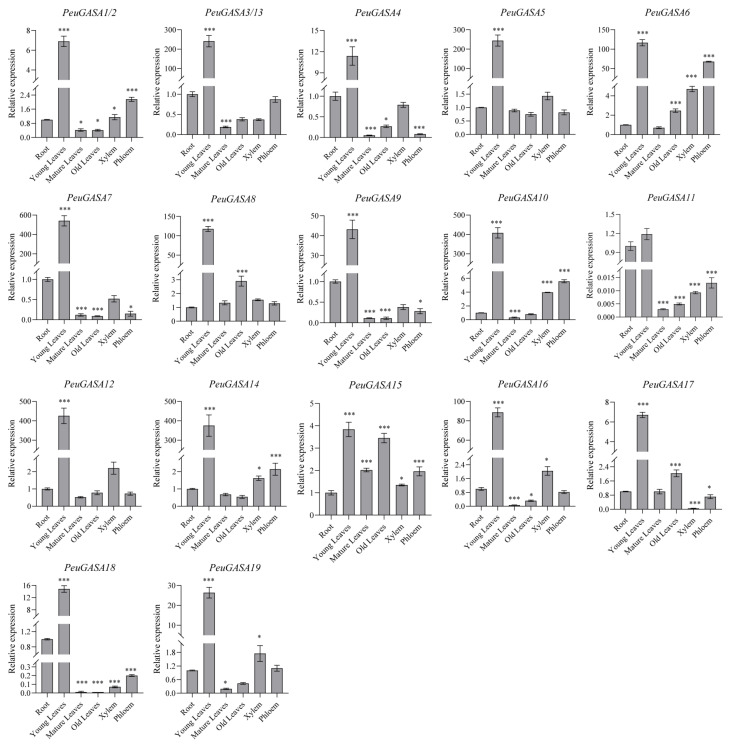
Expression patterns of *GASA* genes in different *P. euphratica* tissues. Data were presented as mean ± standard error (SE). Subsequent multiple comparisons were evaluated based on the least significant difference (LSD) test to calculate *p*-values (Asterisks denoted significant differences: * *p* ≤ 0.05; *** *p* ≤ 0.01).

The expression patterns of *GASA* genes in different tissues of *P. trichocarpa* were visualized in Supplementary Figure S2. *PtrGASA12* was identified unexpressed in the selected dataset. The expression of *PtrGASA3* and *PtrGASA11* were hardly detected in *P. trichocarpa*. *GASA* genes in *P. trichocarpa* were highly expressed mainly in the stages of buds dormant, buds pre-chilling, leaves young expanding, leaves freshly expanded, flowers dormant and flowers expanding. And the expression levels decreased with leaf extension and flower opening. *PtrGASA9*, *PtrGASA16*, *PtrGASA18*, and *PtrGASA21* were highly expressed in wood, *PtrGASA18* and *PtrGASA7* were highly expressed in root. Similar to the expression patterns of *PtrGASA*s, the expression levels of *PeuGASA* genes in young leaves were significantly higher than those in other tissues and organs, while the expression levels of *PeuGASA* genes in mature leaves were significantly lower than these in other tissues and organs (Figure 6). Compared with mature leaves, the expression levels of *PeuGASA4*, *PeuGASA6*, *PeuGASA8*, *PeuGASA10*, *PeuGASA11*, *PeuGASA15*, *PeuGASA16*, and *PeuGASA17* were higher in old leaves. *PeuGASA*s were also expressed in the vascular tissues. *PeuGASA1/2*, *PeuGASA6*, *PeuGASA11*, *PeuGASA14*, and *PeuGASA15* were highly expressed in phloem, and *PeuGASA1/2*, *PeuGASA6*, *PeuGASA10*, *PeuGASA14*, *PeuGASA15*, *PeuGASA16*, and *PeuGASA19* were highly expressed in xylem. Except for *PeuGASA8* and *PeuGASA15*, the expression levels of *PeuGASA*s in roots were not as high as that in young leaves, but mostly higher than that in mature leaves.

### 2.5. Responses of GASAs to Exogenous Hormones in P. euphratica Leaves

**Figure 7:**
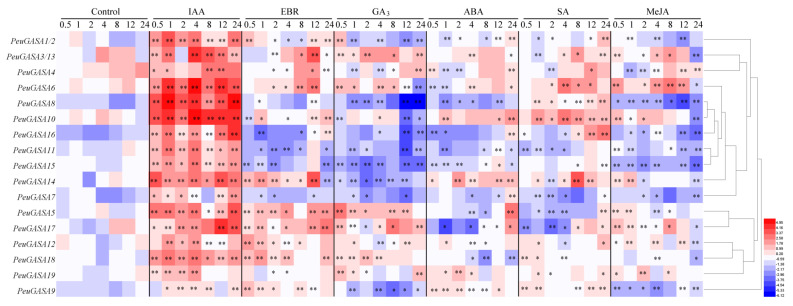
Expression analysis of *PeuGASA* genes in response to SA, ABA, MeJA, EBR, GA_3_ and IAA treatments. Numbers are the time (h) of treatment. The heat map was based on the calculation of three replicates of qRT-PCR data and the transformation of log2 algorithm. Multiple comparisons were evaluated based on the least significant difference (LSD) test to calculate *p*-values (Asterisks denoted significant differences: * *p* ≤ 0.05; ** *p* ≤ 0.01).

To investigate the responses of *GASA* genes to exogenous hormones in *P. euphratica* leaves, the transcriptional levels of candidate genes were studied (Figure 7). After treatment with the stress hormone abscisic acid (ABA), the expression levels of *PeuGASA8*, *PeuGASA11*, *PeuGASA15*, *PeuGASA17*, and *PeuGASA18* were down-regulated, the expression levels of *PeuGASA1*/*2* and *PeuGASA16* were initially down-regulated and up-regulated subsequently, but the expression levels of *PeuGASA9*, *PeuGASA10* and *PeuGASA14* were up-regulated. After being treated with salicylic acid (SA), the expression levels of *PeuGASA6*, *PeuGASA10*, and *PeuGASA14* were significantly up-regulated and the expression levels of *PeuGASA5*, *PeuGASA7*, *PeuGASA11*, and *PeuGASA17* were significantly down-regulated. The expression levels of *PeuGASA1/2*, *PeuGASA4*, *PeuGASA8*, *PeuGASA9*, and *PeuGASA15* were down-regulated in the leaves of *P. euphratica* treated by methyl jasmonate (MeJA), while the expression levels of *PeuGASA6*, *PeuGASA10*, *PeuGASA14*, *PeuGASA16*, and *PeuGASA17* were initially up-regulated and then down-regulated. After auxin (IAA) treatment, the expression levels of *PeuGASA* genes in *P. euphratica* leaves were sharply up-regulated, even increased dozens of times. The expression levels of *PeuGASA1/2*, *PeuGASA5*, *PeuGASA6*, *PeuGASA9*, *PeuGASA12, PeuGASA14*, and *PeuGASA17* were up-regulated in *P. euphratica* leaves after 24-epibrassinolide (EBR) treatment, while *PeuGASA11* and *PeuGASA15* were down-regulated. After gibberellin (GA_3_) treated, the expressions *PeuGASA1/2*, *PeuGASA4*, *PeuGASA8*, *PeuGASA9*, *PeuGASA10*, *PeuGASA11*, *PeuGASA14*, and *PeuGASA15* were inhibited, while the expressions of *PeuGASA3/13*, *PeuGASA5*, *PeuGASA6*, *PeuGASA12*, *PeuGASA17*, and *PeuGASA19* were induced.

### 2.6. Response of GASAs to Stresses in Populus Leaves

**Figure 8:**
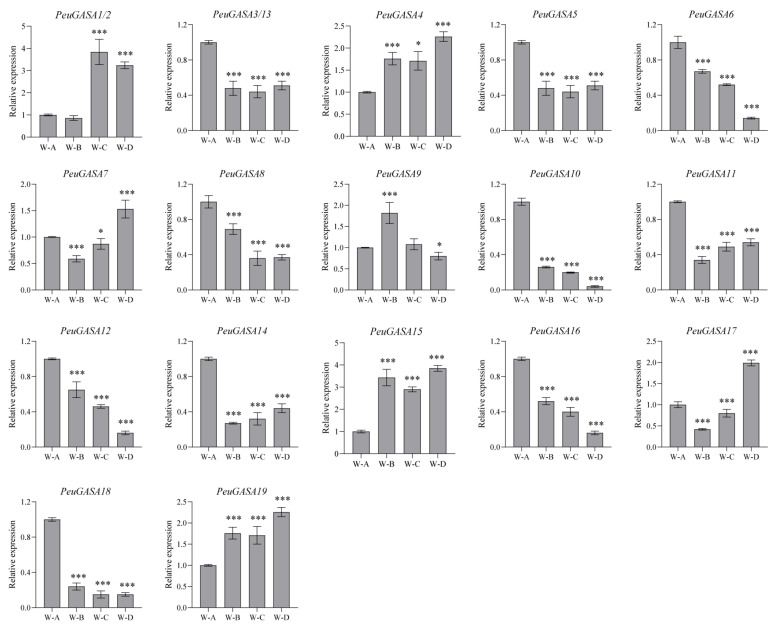
Expression analysis of *PeuGASA* genes in response to drought stress. W-A, W-B, W-C, and W-D respectively represented four drought treatments with different soil volumetric water content (soil-VWC): control (43 ± 1% soil-VWC), mild drought (33 ± 1% soil-VWC), moderate drought (23 ± 1% soil-VWC) and severe drought (13 ± 1% soil-VWC). Data were presented as mean ± standard error (SE). Subsequent multiple comparisons were evaluated based on the least significant difference (LSD) test to calculate *p*-values (Asterisks denoted significant differences: * *p* ≤ 0.05; *** *p* ≤ 0.01).

To further explore the function of *GASA*, *GASA* genes respond to stresses in *Populus* leaves had been investigated (Figure 8 and Supplementary Figure S3). No changes were detected in the expressions of *PtrGASA3*, *PtrGASA5*, *PtrGASA11*, and *PtrGASA15* after drought, beetle damage, and mechanical damage (Supplementary Figure S3). No changes in the expression levels of *PtrGASA7*, *PtrGASA13*, and *PtrGASA21* were detected after beetle damage and mechanical damage. After beetle damage and mechanical damage, the expressions of most *PtrGASA* genes were strongly inhibited, while the expression of *PtrGASA9* was strongly induced. Mechanical damage slightly induced the expression of *PtrGASA19*. Another exception was that the expression of *PtrGASA6* was inhibited by beetle damage but induced by mechanical damage. Compared with control group, the expressions of *PtrGASA2*, *PtrGASA4, PtrGASA6*, *PtrGASA7*, *PtrGASA8*, *PtrGASA9*, *PtrGASA13*, *PtrGASA19*, and *PtrGASA21* were induced, while the expressions of *PtrGASA1*, *PtrGASA10*, *PtrGASA14*, *PtrGASA16*, *PtrGASA17*, *PtrGASA18*, and *PtrGASA20* were inhibited in drought stress. Similar to *P. trichocarpa*, the expressions of *GASA* genes in *P. euphratica* leaves responded to drought stress. Drought stress induced the expression of *PeuGASA1*, *PeuGASA2*, *PeuGASA4*, *PeuGASA15*, and *PeuGASA19*, but inhibited the expression of *PeuGASA3*, *PeuGASA5*, *PeuGASA6*, *PeuGASA8*, *PeuGASA10*, *PeuGASA11*, *PeuGASA12*, *PeuGASA13*, *PeuGASA14*, *PeuGASA16*, and *PeuGASA18* (Figure 8).

### 2.7. Identification of cis-Acting Elements in the Promoter Region of GASA Genes

**Figure 9:**
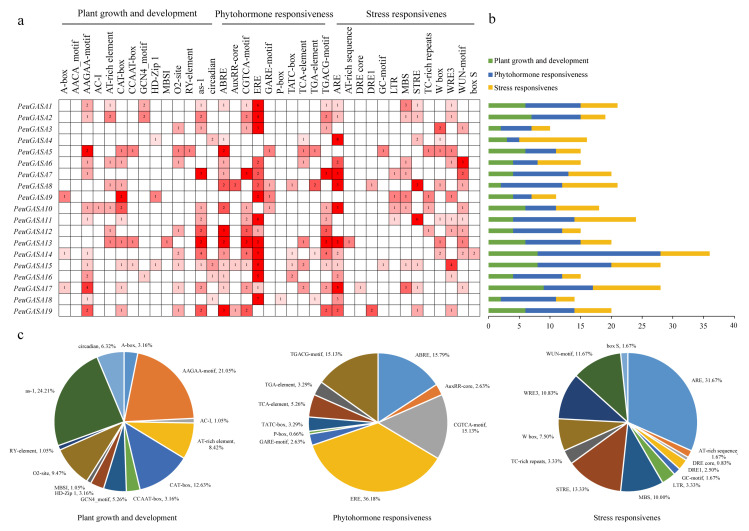
The *cis*-acting elements of *PeuGASA* genes. (**a**) Numbers and gradient red colors indicate the number of *cis*-acting elements; (**b**) Color-coded histograms indicate the number of *cis*-acting elements of genes in each category; (**c**) Pie charts show the proportion of different *cis*-acting elements in each category.

To predict the potential biological functions of the *GASA* genes, the *cis*-acting elements of the promoters were analyzed. The *cis*-acting elements in the promoters of *GASA* genes related to plant growth and development, hormone response, and stress response were displayed (Figure 9 and Supplementary Figures S4 and S5). The *cis*-acting regulatory element related to meristem expression, CAT-box, was found in about half of *GASA* promoters. O2-site, the *cis*-acting regulatory element involved in zein metabolism regulation, were found in eight *PeuGASA* promoters, whereas in four *PtrGASA* promoters. Almost all promoters of *GASA* genes had the *cis*-acting element ABRE involved in the abscisic acid responsiveness and the *cis*-acting element ERE involved in ethylene responsiveness, especially ERE elements were abundant. The *cis*-acting elements ABRE in the *GASA* gene promoters of *P. trichocarpa* were 1.7 times that of *P. euphratica*. About half of *GASA* gene promoters had *cis*-acting elements involved in gibberellin-responsiveness (52.39% in *P. trichocarpa*, 42.11% in *P. euphratica*). Only one P-box element (gibberellin-responsive element) was found in the *GASA* gene promoters of *P. euphratica* (*PeuGASA18*), while eight P-box elements were found in the seven *GASA* gene promoters of *P. trichocarpa*. The *cis*-acting elements involved in mechanical damage responsiveness in the *GASA* gene promoters of *P. euphratica* were more than those of *P. trichocarpa*, totally accounting for 30%. The *cis*-acting element LTR involved in low-temperature responsiveness and TC-rich repeats involved in defense and stress responsiveness in the *GASA* gene promoters of *P. trichocarpa* were significantly more than those of *P. euphratica*. Besides, *GASA* gene promoters with *cis*-acting elements involved in stress responsiveness, STRE, accounted for 57.89% in *P. trichocarpa*, while 38.10% in *P. euphratica*.

### 2.8. Analysis of PeuGASA15 Promoter Activity

**Figure 10:**
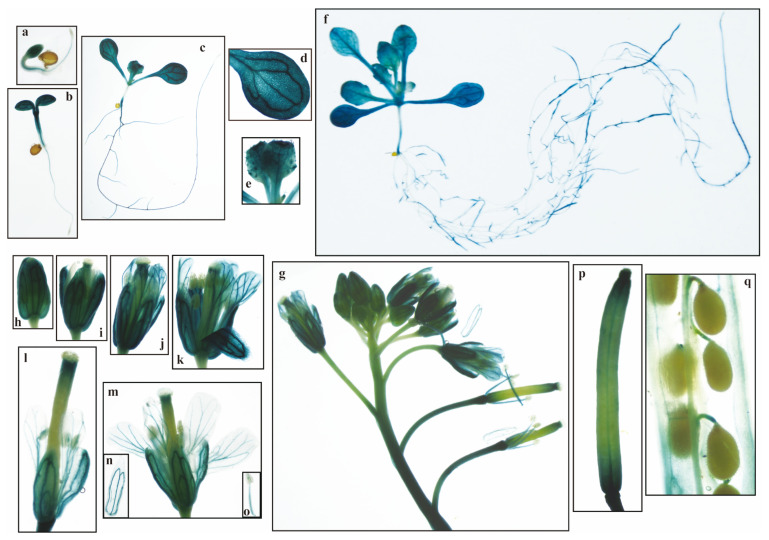
GUS activity of the *PeuGASA15* promoter. (**a**) 3-day-old plant; (**b**) 4-day-old plant; (**c**) 7-day-old plant; (**d**,**e**) Cotyledons and young leaves of 7-day-old plant; (**f**) 14d-day-old plant; (**g**) inflorescence; (**h**–**k**) Expanding flowers; (**l**) Fading flowers; (**m**) Expanded flower; (**n**) Petal; (**o**) Stamen; (**p**,**q**) Silique.

**Figure 11:**
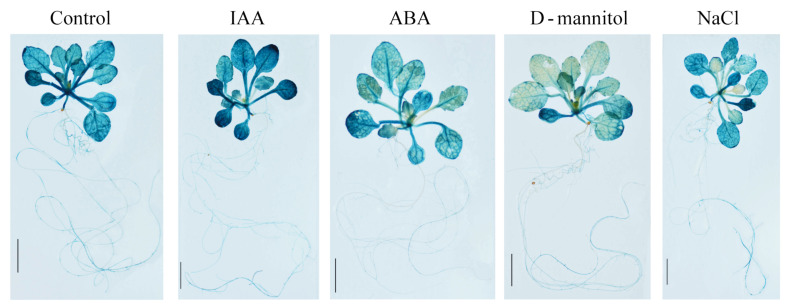
Three-week-old ProGASA15::GUS transgenic *Arabidopsis* seedlings growing on 1/2 MS AGAR plates were transplanted into medium without or containing 300 mM mannitol, 150 mM NaCl, 20 μM ABA, 20 mg/L IAA, respectively.

Compared with other *PeuGASA* genes, *PeuGASA15* was highly expressed in multiple tissues and its expression responded to hormones and drought stress indicating that *PeuGASA15* might be actively involved in various stress regulatory networks, hormone signaling, and metabolic pathways in the *GASA* gene family. To verify the temporal and spatial expression patterns of *PeuGASA*s, *PeuGASA15* was selected as the representative gene. GUS activity in transgenic *Arabidopsis* at different growth and development stages was detected by histochemical staining (Figure 10, Supplementary Figure S6). In the initial stage of vegetative growth of pGASA15::GUS transgenic *Arabidopsis*, strong GUS activity was detected in cotyledons, leaves, hypocotyls, roots, especially in their vascular tissues (Figure 10a–e, Supplementary Figure S6c). GUS activity detected in cotyledons was still strong at the peak of vegetative growth (Figure 10f). Strong GUS activity was detected in several guard cells of leaves (Supplementary Figure S6b). In young leaves unexpanded, GUS activity was mainly detected in leaf tips and vascular bundles. GUS activity was not uniform in roots, and strong GUS activity was detected in the initial and growing parts of lateral roots and the tip parts of taproots (Supplementary Figure S6c–g). During the reproductive growth stage, GUS activity increased with the development of the flower, and then gradually decreased. GUS activity was detected in sepals, petals, stigmas and stamens of flowers, especially in their vascular bundles (Figure 10h–m). GUS activity in the parenchyma tissues and vascular bundles of pGASA15::GUS transgenic *Arabidopsis* flower stems was detected (Supplementary Figure S6h–i). Besides, GUS activity was detected in the funiculus of the dissected silique (Figure 10q). GUS activity of 3-week-old transgenic seedlings was significantly increased in 1/2 MS agar medium with 20 mg/L IAA for 4 h and decreased in 1/2 MS ager medium with 20 μM ABA for 24 h (Figure 11). The expression patterns of pGASA15::GUS were consistent with the result of qRT-PCR. Mannitol strongly inhibited *PeuGASA15* expression in the leaves of transgenic *Arabidopsis*. NaCl also had a strong inhibitory effect on *PeuGASA15* expression in the unexpanded leaves of pGASA15::GUS transgenic *Arabidopsis* (Figure 11).

## 3. Discussion

GASAs, a class of cysteine-rich small peptides, consists of a putative signal peptide at the N-terminal and a conserved GASA domain containing 12 cysteine residues at the C-terminal. GASA proteins play an important role in many aspects of plant growth and development, biotic and abiotic stresses responses, and hormonal signaling [8,13,15,17,18,19,27,32]. Extensive studies about the biological functions and bioinformatics analysis of *GASA* genes have been reported in many plants, but little information concerning *P. euphratica* has been known. In this study, 21 and 19 candidate *GASA* genes were identified in *P. trichocarpa* and *P. euphratica*, respectively, and the bioinformatics and expression patterns of these genes were analyzed. There were candidate *GASA* genes in *P. trichocarpa*, but no corresponding homologous genes have been found in *P. euphratica*. This may be because the genome of *P. euphratica* was not as well sequenced as that of *P. trichocarpa*. It is also possible that these extra members formed in *P. trichocarpa* after two species differentiated, or that these ancestral sequences were lost in *P. euphratica* [43].

Like potato and apple, the *GASA* genes were distributed unevenly on several chromosomes in *P. trichocarpa* (Figure 1), while they were distributed on all chromosomes in *Arabidopsis* [15,44]. The proteins encoded by the *GASA* genes identified were low molecular weight proteins, which were consistent with the results of *Arabidopsis* [29]. GASA proteins were mainly predicted to be signal peptides secreted through the classical secretion pathway and localized in extracellular. At present, numerous experiments in vivo have proved that most of the GASA proteins are localized in the cell wall or extracellular region [17,45,46,47,48]. *GASA* genes have not been found in *Physcomitrella patens* whose whole genome has been sequenced, possibly because *GASA* genes appeared in vascular plants and originated from pteridophytes [9]. Due to the difference in occurrence and distance between the motifs, these GASA domains were distributed discretely in the phylogenetic tree and mainly divided into three clusters (Figure 4). In subfamily B, there were successive and independent interspecific clades, suggesting that the GASA domains of subfamily B might be relatively ancient and conserved among species (Figure 2). The GASA domains of *S. moellendorffii* were not found in the subfamily A, suggesting that this subfamily might have evolved late and originated after pteridophytes. *SM00035G00570* separated early from subfamily C, indicating that its evolution was completed earlier and might be the origin of this subfamily. Compared with other species, GASAs of poplar and *Arabidopsis* had relatively higher substitution rates and closer genetic relationships.

Gene replication drives the evolution of genomes and genetic systems and contributes to the diversity of gene structure and function in gene families. WGD and TD are considered to be the two main causes of plant gene family expansion [49]. The number of WGD genes decreased significantly with the prolongation of replication event time, while those of TD genes did not decrease with time. Although the number of derived duplicated genes decreased exponentially, certain WGD gene pairs were still retained in *P. trichocarpa* and *P. euphratica* (Table 2). WGD genes in subfamily B degenerated into nonfunctional or disappeared in the long-term evolution which might lead to the deletion of *GASA* genes in *P. euphratica*. WGD genes played a dominant role in the evolution of poplar *GASA* genes in subfamily B. Different from other species with relatively uniform distribution in each subfamily, the GASAs of *P. euphratica* and *P. trichocarpa* mostly belong to subfamily A due to the gene expansion. *GASA* genes in subfamily A retained the conserved WGD genes and evolved new TD genes. TD genes provided variation for adapting to changing environments and tended to evolve subfunctions or new functions involved in plant defense, while WGD genes were relatively conserved [50]. *GASA* genes in subfamily C neither retained WGD genes nor generated TD genes. The expansion of subfamily C genes might be mainly due to transposition events.

The *cis*-acting elements predicted in the *GASA* gene promoters of *Populus* are roughly identical (Figure 9 and Supplementary Figures S4 and S5). However, there were significant differences in the number of *cis*-acting elements involved in response to abscisic acid, gibberellin, mechanical damage, low temperature, drought between the promoters of *GASA* gene families in *P. trichocarpa* and *P. euphratica*. The expression patterns of the *P. euphratica GASA* gene family were roughly consistent with those of *P. trichocarpa*. After drought treatment, the expression trends of WGD gene pairs, *PeuGASA9* and *PeuGASA15*, *PeuGASA6* and *PeuGASA13* were consistent respectively. The expression trends of another WGD gene pair, *PeuGASA10* and *PeuGASA19*, were opposite (Figure 8). This tendency was identical with their homologous genes in *P. trichocarpa*. And there were almost similar expression trends between WGD pair genes after hormonal treatment in *P. euphratica* mature leaves. Whereas, the expression trends of TD gene pairs were consistent in *P. trichocarpa* which did not share in *P. euphratica* under drought stress. Partial divergence also appeared in the expression trends of TD gene pairs after hormonal treatment. The long-term adaptation to the complex terrestrial environments might lead to the differences in *GASA* genes expression between *P. euphratica* and *P. trichocarpa*.

GASA1, as a representative gene of GA signal response and transduction, has been mentioned in many reports [51,52,53,54,55,56]. GASA1 is involved in cell elongation, reproductive growth, leaf senescence, biological and abiotic stress regulatory networks [34,37,52,53,56,57]. GASA1 expression also responded to abscisic acid, brassinosteroid and auxin [29,58,59]. AtGASA1, the homolog of PeuGASA15, has been reported to weakly interact with VIT by phage display technology [60]. VIT, also named TETRATRICOPEPTIDE-REPEAT THIOREDOXIN-LIKE 1 (TTL3), plays an important role in brassinosteroid and auxin signaling, and is required for osmotic stress tolerance [60,61,62,63,64,65]. Consistent with *Arabidopsis*, IAA induced *PeuGASA15* expression and mannitol significantly inhibited *PeuGASA15* expression in the leaves of pGASA15: GUS transgenic leaves (Figure 11). GUS gene was highly expressed in various organs of the whole pGASA15: GUS transgenic plant, especially in vascular tissues (Figure 11 and Supplementary Figure S6). Vascular tissues are present in vascular plants, and their main functions are to transport water, inorganic salts, and nutrients and to provide mechanical support for plants [66]. The TTL gene family, like the *GASA* gene family, is endemic to land plants. VIT also interacted with VH1/BRL2 which plays a role in vascular development [60]. This finding might prove that PeuGASAs are involved in the growth and development of vascular tissues, and provide evidence that the origin of the *GASA* gene family in vascular plants might be related to their functions.

The expression patterns of genes in different tissues or organs indicate the possible biological functions of these genes [67]. The expression levels of *PeuGASA* genes in young leaves were relatively high but decreased with leaf maturity, suggesting that *PeuGASA* genes might be involved in leaf growth and development (Figure 6) [20]. Auxin is mainly distributed in the growth vigorous and young tissues of plants [68,69]. *GASA* genes were mainly expressed in the young tissues of *Populus*, and the expression of *PeuGASA* genes could be induced by auxin, indicating that *GASA* genes might play a role in the auxin signal pathway. Gibberellin regulates embryo development, seed germination, stem and root elongation, and flower organ development [70,71]. *GASA* genes are GA target genes located downstream of the GA signaling pathway [29]. The expression of the *GASA* gene family was strongly inhibited or induced by gibberellin (Figure 7). It has been reported that the *GASA* gene family was involved in seed germination, shoot and stem elongation, lateral root formation, flowering and flower development, fruit development, and ripening [15,16,17,18,19,21,22,23,24,25,28]. *PeuGASA*s were also induced or inhibited by EBR, ABA, SA, and MeJA, indicating that *GASA*s might be extensively involved in the complex plant hormonal signal transduction network [26]. The *cis*-acting elements responding to stress hormones and stresses in the promoters of *PtrGASA* and *PeuGASA* genes and the expression of *PtrGASA* and *PeuGASA* genes was affected by drought, mechanical damage, or other stresses, indicating that PtrGASAs and PeuGASAs play roles in the stress environments. In conclusion, *PeuGASA*s may be involved in the growth and development, plant hormone responses and signal transduction, and responses to abiotic stresses.

## 4. Materials and Methods

### 4.1. Identification of GASA Family Genes in P. trichocarpa and P. euphratica

The Hidden Markov Model (HMM) profile of the GASA domain (PF02704) from the Pfam database was used as a query and the putative GASA protein sequences were identified by the HMMER searching against the *P. euphratica* genome. These sequences were further confirmed by using Pfam 34.0 database (http://pfam.xfam.org/, accessed on 13 July 2021) and blasted by using Protein BLAST in the NCBI database (https://blast.ncbi.nlm.nih.gov/Blast.cgi, accessed on 15 July 2021). The genomic, transcript, CDS, peptide and, 2000 bp upstream of the translational start site (ATG) promoter region sequences in *P. euphratica* were extracted from the NCBI database (https://www.ncbi.nlm.nih.gov/, accessed on 2 September 2021). The *GASA* genes in *P. trichocarpa* were obtained from the Phytozome 12.0 database (https://phytozome.jgi.doe.gov/pz/portal.html#!info?alias=Org_Ptrichocarpa, accessed on 18 July 2021) and confirmed by the above method. The *PeuGASAs* and *PtrGASAs* were named according to their position on the scaffolds and chromosomes respectively. The *PeuGASAs* homologous genes in *P. trichocarpa* were blasted by Protein BLAST and finally confirmed by MEGA 6.0 (Tokyo Metropolitan University, Hachioji, Japan) [72]. The gene position of *PtrGASA*s and chromosome size were obtained from Phytozome 12.0 and visualized by the TBtools (South China Agricultural University, Guangzhou, China). The *cis*-acting elements of promoters were predicted using PlantCARE (http://bioinformatics.psb.ugent.be/webtools/plantcare/html/, accessed on 5 September 2021) and classified by reference [73,74,75]. The results were subsequently visualized by Morpheus (https://software.broadinstitute.org/morpheus/, accessed on 8 September 2021) and Excel (Microsoft, Redmond, WA, USA).

### 4.2. Physical and Chemical Properties, Subcellular Localization, Multiple Alignments and Collinearity Analysis

Physical and chemical parameters of GASAs were computed by ProtParam (https://web.expasy.org/protparam/, accessed on 15 August 2021). The subcellular localization sites of GASAs were predicted by WoLF PSORT (https://wolfpsort.hgc.jp/, accessed on 16 August 2021) and TargetP 1.1 Server (http://www.cbs.dtu.dk/services/TargetP-1.1/index.php, accessed on 17 August 2021). The PeuGASA and PtrGASA protein sequences were multiple aligned by DNAMAN Version 7, and the multiple sequences alignment was visualized with WebLogo version 2.8.2 (http://weblogo.berkeley.edu/logo.cgi, accessed on 12 August 2021). The gene structure map of *GASA* genes was drawn by using the gene structure visualization server GSDS2.0 (http://gsds.gao-lab.org/, accessed on 11 September 2021). GASA proteins sequences were submitted to MEME Suite 5.3.3 (https://meme-suite.org/meme/tools/meme, accessed on 12 September 2021) to predict the conserved motifs with default settings for parameters (the maximum number of motifs set to 5). Candidate GASA domains were aligned with the default parameters using MEGA 6.0 [72]. The phylogenetic trees were constructed based on the neighbor-joining method with a bootstrap test of 1000 replicates. The occurrence replication events of *GASA* genes in *P. trichocarpa* were analyzed and visualized by MCScanX (University of Georgia, Athens, GA, USA) [76], blast, and TBtools [77]. The above software was also used for the collinearity analysis of *GASA* genes in *Arabidopsis* and *P. trichocarpa* genome.

### 4.3. Plant Materials and Sample Collections

One-year-old *Populus euphratica* seedlings from Xinjiang Uygur Autonomous Region of China were cultivated in the plastic greenhouse. Each pot contained 3–5 seedlings. The potted seedlings were grown for about 3–4 months under environmentally controlled conditions, and three potted seedlings with the same growth were selected for each treatment. The selected seedlings were treated with 100 μΜ GA3, 200 μM ABA, 100 mg/L IAA, 10 μM EBR, 100 μM SA, and 100 μM MeJA respectively. The control group was an aqueous solution containing 0.1% anhydrous ethanol and 0.02% (*v*/*v*) Triton X-100 [78,79]. Sprayed the whole plant evenly until the leaves are about to drip. Then collected the mature leaves of the seedlings at 0, 0.5, 1, 2, 4, 8, 12, and 24 h after treatment. Drought treatment of seedlings was performed according to the method of Tang et al. [37]. All the collected samples were immediately frozen in liquid nitrogen and stored at −80 °C.

### 4.4. RNA Extraction, cDNA Synthesis, and qRT-PCR Analysis

The total RNA of samples was extracted using EasySpin Plus Plant RNA Kit (Aidlab Biotech, Beijing, China). The first cDNA strand was synthesized by FastQuant RT Kit (with gDNase, Tiangen, Beijing, China). The specific primers were designed by Primer Premier 6.0 and examined by NCBI Primer-BLAST (https://www.ncbi.nlm.nih.gov/ tools/primer-blast/, accessed on 15 July 2021) (Supplementary Table S4). The selection of reference genes of *P. euphratica* was based on the research of Wang et al. [80,81]. Real-time PCR was run with the CFX96 Touch™ instrument (Bio-Rad, Hercules, CA, USA) to detect the chemical SYBR Green. The established reaction system was as follows: 10 μL 2× SuperReal Premix Plus (Tiangen), 0.6 μL each forward and reverse primers (10 μM), 1 μL diluted cDNA template, and the finally RNase-free ddH_2_O was added until the total amount was 20 μL. The reaction procedures were: 95 ℃ for 15 min, 45 cycles of 10 s at 95 ℃ and 30 s at 60 ℃. The melting curve analysis was performed immediately after the PCR reaction, i.e., fluorescence intensity was measured for each degree increase from 60 ℃ to 95 ℃. The relative template abundance in each PCR expansion mixture was calculated by the 2^−ΔΔCT^ method [82]. Three biological replicates were used for gene expression analysis, and the expressions of the control samples were set to 1 for normalization. SPSS (IBM, Armonk, NY, USA) was used for statistical analysis, and the LSD test was used to calculate the *p* value. Finally, the results were presented as histograms by GraphPad Prism 8 software (GraphPad, San Diego, CA, USA) or heat maps by Heml software (University of Science and Technology, Wuhan, China).

### 4.5. Subcellular Localization of PeuGASA Proteins

The full-length coding sequences of *PeuGASA* genes without stop codon were amplified by PCR gene using gene-specific primers (Supplementary Table S5). To express *PeuGASA*-GFP fusion proteins, the products were cloned between the Super promoter and GFP gene in the pSUPER1300 (+) vector [83]. The confirmed construct fusion vectors were introduced into *Agrobacterium tumefaciens* strain GV3101 and transformed into *Nicotiana benthamiana* leaves via *Agrobacterium*-mediated gene transformation for transient expression [84]. GFP fusion protein GFP signals were detected 2 days after infiltration using Leica fluorescence microscope DM2500 (Leica Microsystems, Wetzlar, Germany).

### 4.6. Arabidopsis Culture, Construction and Transformation of Vector, and GUS Activity Assay

*Arabidopsis* was planted on 1/2MS medium containing 3% (*w*/*v*) sucrose and 0.6% (W/V) ager at 23 °C with white light fluorescence and photoperiod of 16/8 h (Light/dark). Seven days after seed germination, *Arabidopsis* was transplanted into vegetative soil and cultured in an incubator to complete the whole growth cycle. A binary vector was constructed using the cloned *PeuGASA15* promoter. The fragments were digested with BamHI and EcoRI enzymes and inserted into the binary vector pCAMBIA1391. The vectors were introduced into *Agrobacterium tumefaciens* strain GV3101 and transformed into *Arabidopsis* by inflorescence immersion [85]. The transformants were screened on 1/2MS medium supplemented with 25 mg/L hygromycin. T2 was used for subsequent experiments. For histochemical GUS analysis, the prepared materials were soaked in GUS staining solution and incubated overnight at 37 ℃. Then the leaves were decolorized with 70% ethanol and observed under a Leica microscope and an OLYMPUS SZ61 stereomicroscope (Olympus Corporation, Hachioji, Japan).

## Figures and Tables

**Table 1:** Identification of *GASA* gene family in *P. euphratica*.

(a) Identification in the Protein Database Reported by Ma et al.					
Protein ID	Transcript ID	Gene	Gene Description	CDS (bp)	Peptide (aa)
XP_011001772.1	XM_011003470.1	LOC105108948	gibberellin-regulated protein 9-like	333	110
XP_011015007.1	XM_011016705.1	LOC105118698	gibberellin-regulated protein 9-like	333	110
XP_011016723.1	XM_011018421.1	LOC105120240	snakin-2-like	330	109
XP_011021371.1	XM_011023069.1	LOC105123460	protein RSI-1	288	95
XP_011027306.1	XM_011029004.1	LOC105127637	protein GAST1-like	351	116
XP_011028622.1 /XP_011028623.1	XM_011030320.1 /XM_011030321.1	LOC105128593	snakin-2-like	342	113
XP_011029010.1	XM_011030708.1	LOC105128863	gibberellin-regulated protein 11-like	357	118
XP_011029011.1	XM_011030709.1	LOC105128865	gibberellin-regulated protein 1-like	309	102
XP_011029012.1	XM_011030710.1	LOC105128866	gibberellin-regulated protein 1-like	309	102
XP_011029030.1	XM_011030728.1	LOC105128880	peamaclein-like	267	88
XP_011035751.1	XM_011037449.1	LOC105133442	gibberellin-regulated protein 4	321	106
XP_011038137.1	XM_011039835.1	LOC105135117	gibberellin-regulated protein 2	276	91
XP_011044353.1	XM_011046051.1	LOC105139569	snakin-2-like	330	109
XP_011045095.1	XM_011046793.1	LOC105140101	gibberellin-regulated protein 1-like	312	103
XP_011045096.1	XM_011046794.1	LOC105140102	gibberellin-regulated protein 1-like	309	102
XP_011045097.1	XM_011046795.1	LOC105140103	gibberellin-regulated protein 11-like	297	98
XP_011045379.1	XM_011047077.1	LOC105140300	protein GAST1-like	342	113
XP_011047278.1	XM_011048976.1	LOC105141680	gibberellin-regulated protein 14-like	669	222
**(b) Identification in the Protein Database Reported by Zhang et al.**					
**Protein**	**mRNA**	**Gene**	**CDS (bp)**	**Peptide (aa)**	**BLAST**
GWHPAAYU001024	GWHTAAYU001024	GWHGAAYU001024	477	158	(XP_011047278.1)
GWHPAAYU001570	GWHTAAYU001570	GWHGAAYU001570	267	88	No
GWHPAAYU001972	GWHTAAYU001972	GWHGAAYU001972	342	113	XP_011045379.1
GWHPAAYU008043	GWHTAAYU008043	GWHGAAYU008043	321	106	XP_011035751.1
GWHPAAYU008049	GWHTAAYU008049	GWHGAAYU008049	321	106	XP_011035751.1
GWHPAAYU008363	GWHTAAYU008363	GWHGAAYU008363	288	95	XP_011021371.1
GWHPAAYU012087	GWHTAAYU012087	GWHGAAYU012087	342	113	XP_011028622.1 /XP_011028623.1
GWHPAAYU013554	GWHTAAYU013554	GWHGAAYU013554	330	109	XP_011016723.1
GWHPAAYU016490	GWHTAAYU016490	GWHGAAYU016490	309	102	XP_011029012.1
GWHPAAYU016491	GWHTAAYU016491	GWHGAAYU016491	309	102	XP_011029011.1
GWHPAAYU025413	GWHTAAYU025413	GWHGAAYU025413	351	116	XP_011027306.1
GWHPAAYU029870	GWHTAAYU029870	GWHGAAYU029870	309	102	XP_011045096.1
GWHPAAYU029871	GWHTAAYU029871	GWHGAAYU029871	312	103	XP_011045095.1
GWHPAAYU031162	GWHTAAYU031162	GWHGAAYU031162	276	91	XP_011038137.1

**Table 2:** Duplications of *GASA* genes in *P. euphratica*.

Gene 1	Gene 2	Duplication	E-Value	*Ka*	*Ks*	*Ka*/*Ks*	Selection Pressure
*PtrGASA2*	*PtrGASA11*	WGD	5E-16	0.306574081	1.511177265	0.202871025	Purifying selection
*PtrGASA2*	*PtrGASA13*	WGD	1E-41	0.024592367	0.245409683	0.100209441	Purifying selection
*PtrGASA2*	*PtrGASA16*	WGD	3E-34	0.189006113	0.854755332	0.221123058	Purifying selection
*PtrGASA11*	*PtrGASA13*	WGD	8E-14	0.314370832	1.291132145	0.24348463	Purifying selection
*PtrGASA11*	*PtrGASA16*	WGD	2E-14	0.2812637	1.166421628	0.241133818	Purifying selection
*PtrGASAd13*	*PtrGASA16*	WGD	6E-34	0.175785289	1.055787598	0.166496831	Purifying selection
*PtrGASA7*	*PtrGASA9*	WGD	1E-33	0.189204442	0.883649795	0.214116998	Purifying selection
*PtrGASA14*	*PtrGASA17*	WGD	4E-68	0.041106177	0.225565616	0.182236008	Purifying selection
*PtrGASA5*	*PtrGASA6*	TD	4E-31	0.257142422	0.939998969	0.273556068	Purifying selection
*PtrGASA6*	*PtrGASA7*	TD	1E-34	0.162561762	0.966817469	0.16814111	Purifying selection
*PtrGASA8*	*PtrGASA9*	TD	7E-34	0.320382084	1.907862372	0.167927251	Purifying selection
*PtrGASA11*	*PtrGASA12*	TD	4E-30	0	0	NaN	-
*PeuGASA6*	*PeuGASA13*	WGD	6E-49	0.057332466	0.184523724	0.31070512	Purifying selection
*PeuGASA9*	*PeuGASA15*	WGD	2E-39	0.097292607	0.362138829	0.268661075	Purifying selection
*PeuGASA10*	*PeuGASA19*	WGD	2E-34	0.195458562	0.965443142	0.202454762	Purifying selection
*PeuGASA7*	*PeuGASA8*	TD	2E-32	0.204373366	0.898115959	0.227557883	Purifying selection
*PeuGASA8*	*PeuGASA9*	TD	6E-30	0.185223217	0.962509794	0.192437747	Purifying selection
*PeuGASA14*	*PeuGASA15*	TD	1E-44	0.253240964	0.898681597	0.281791644	Purifying selection
*PeuGASA15*	*PeuGASA16*	TD	3E-35	0.297194132	1.782104232	0.166765853	Purifying selection

## Data Availability

All data supporting the findings of this study are available within the paper and within its Supplementary Materials published online.

## Supplementary Materials

The following are available online at https://www.mdpi.com/article/10.3390/ijms222212336/s1.
