# Supplementary material for: Genome-Wide Comprehensive Analysis of the GASA Gene Family in Populus
Source: Int J Mol Sci. 2021 Nov 15;22(22):12336. doi: 10.3390/ijms222212336 (PMC8624709; doi:10.3390/ijms222212336)
Supplement: Supplementary file 1 [file ijms-22-12336-s001.zip › ijms-1405267-supplementary.pdf]

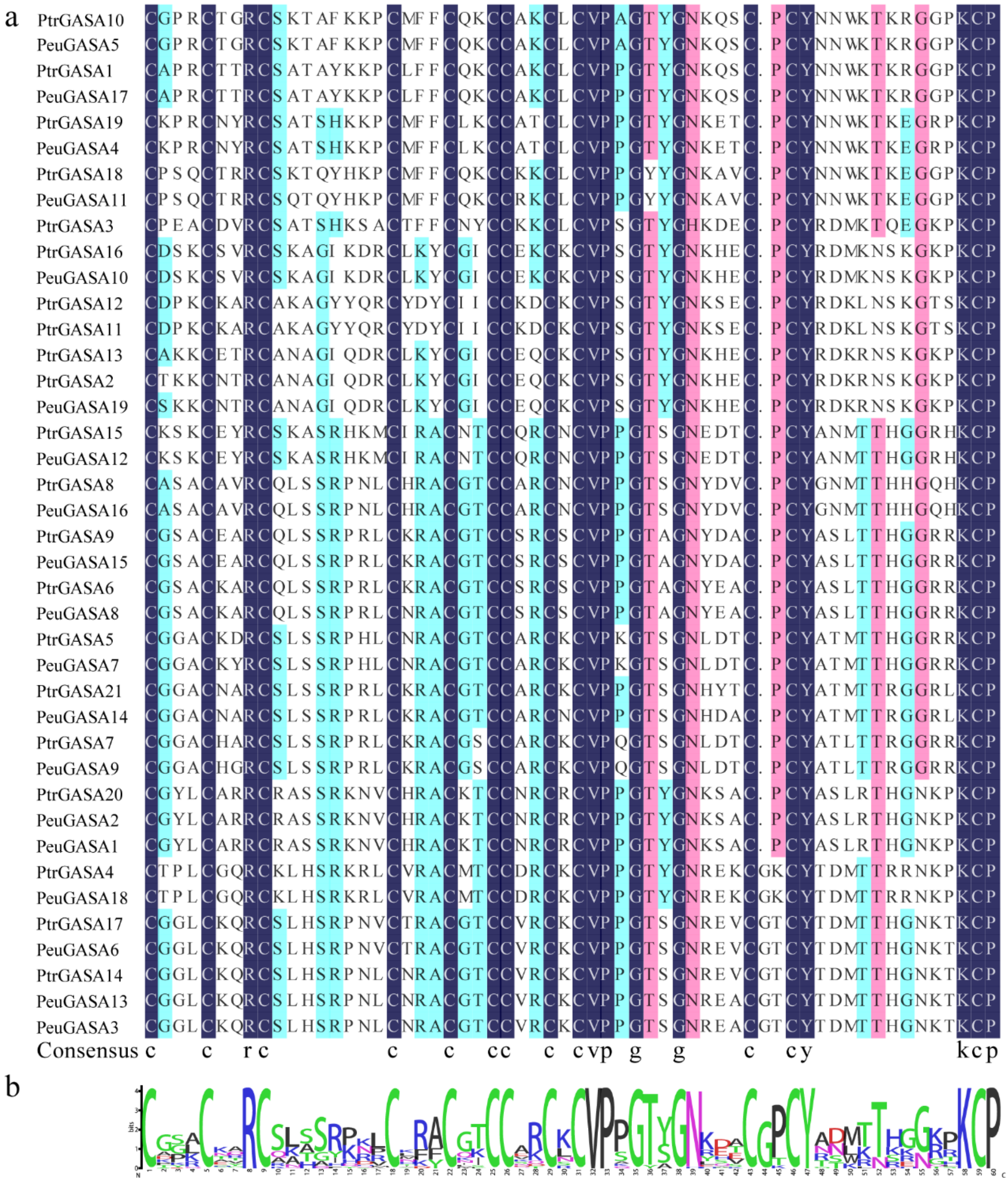

Figure S1. Multiple alignment of *GASA* gene family. (**a**) Multiple alignment of the conserved GASA domains in *P. euphratica* and *P. trichocarpa*; (**b**) Sequence logo of the conserved GASA domains *P. euphratica* and *P. trichocarpa*. Each stack represented the amino acids in their corresponding position.

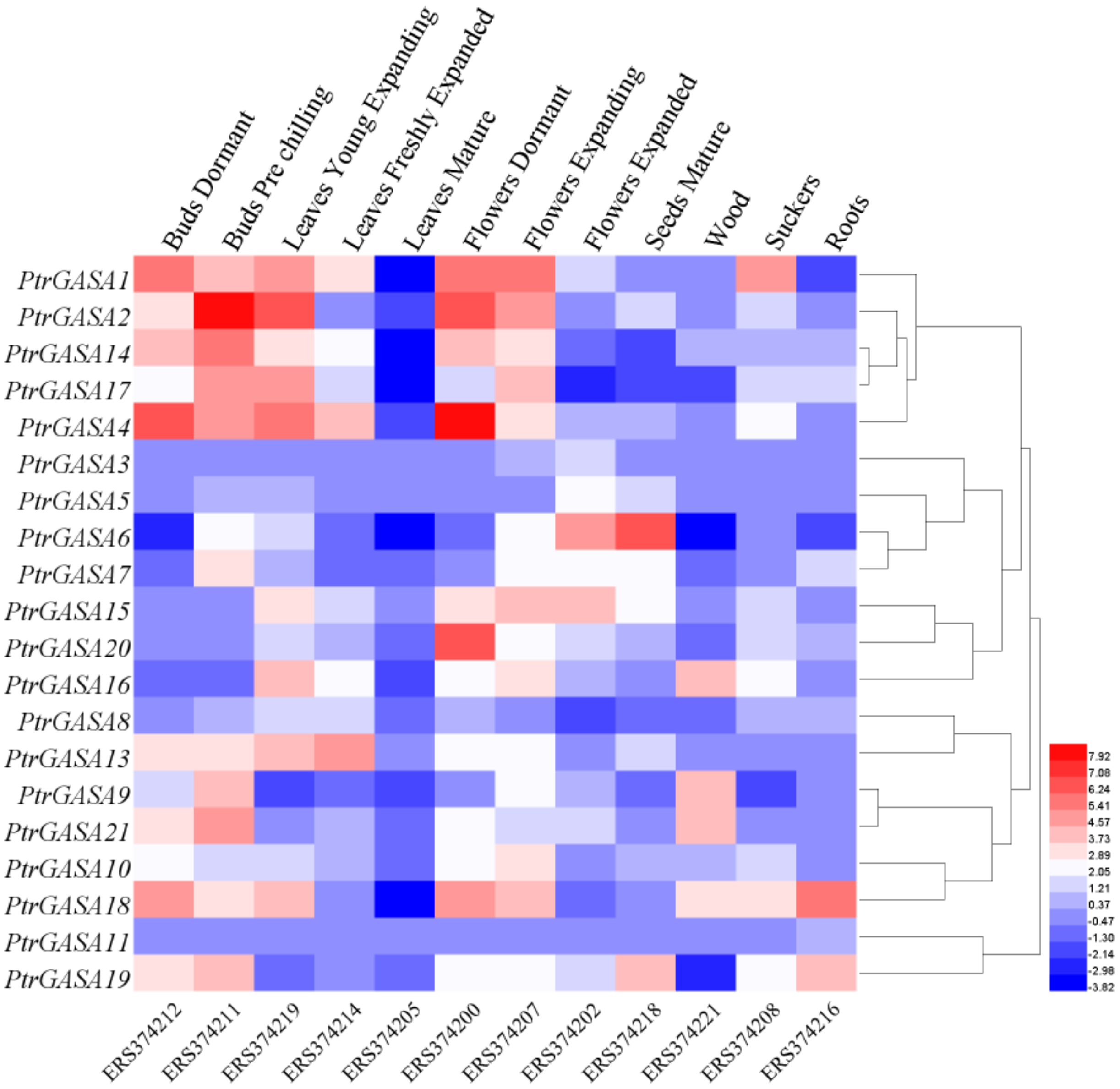

Figure S2. The *PtrGASA*s expression profiles in different tissues. Relative expression profiles are based the online PlantGenIE database.

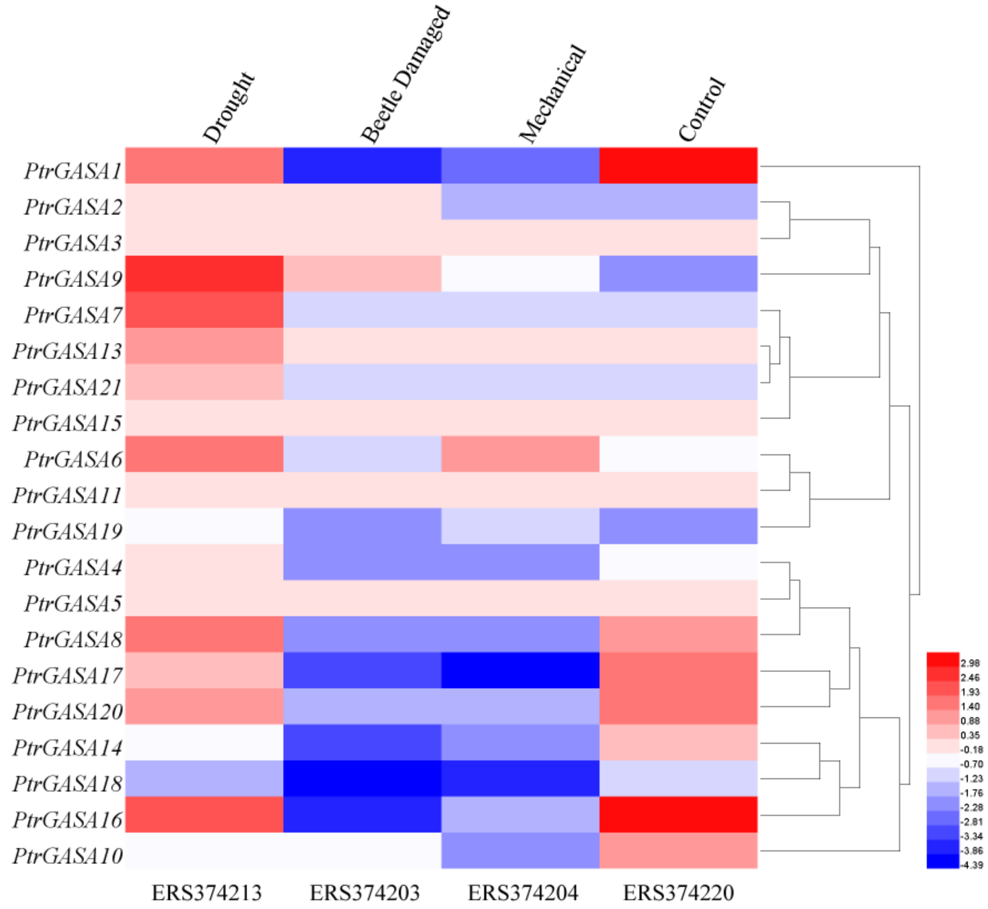

Figure S3. The *PtrGASAs* expression profiles respond to stresses. Relative expression profiles are based the online PlantGenIE database.

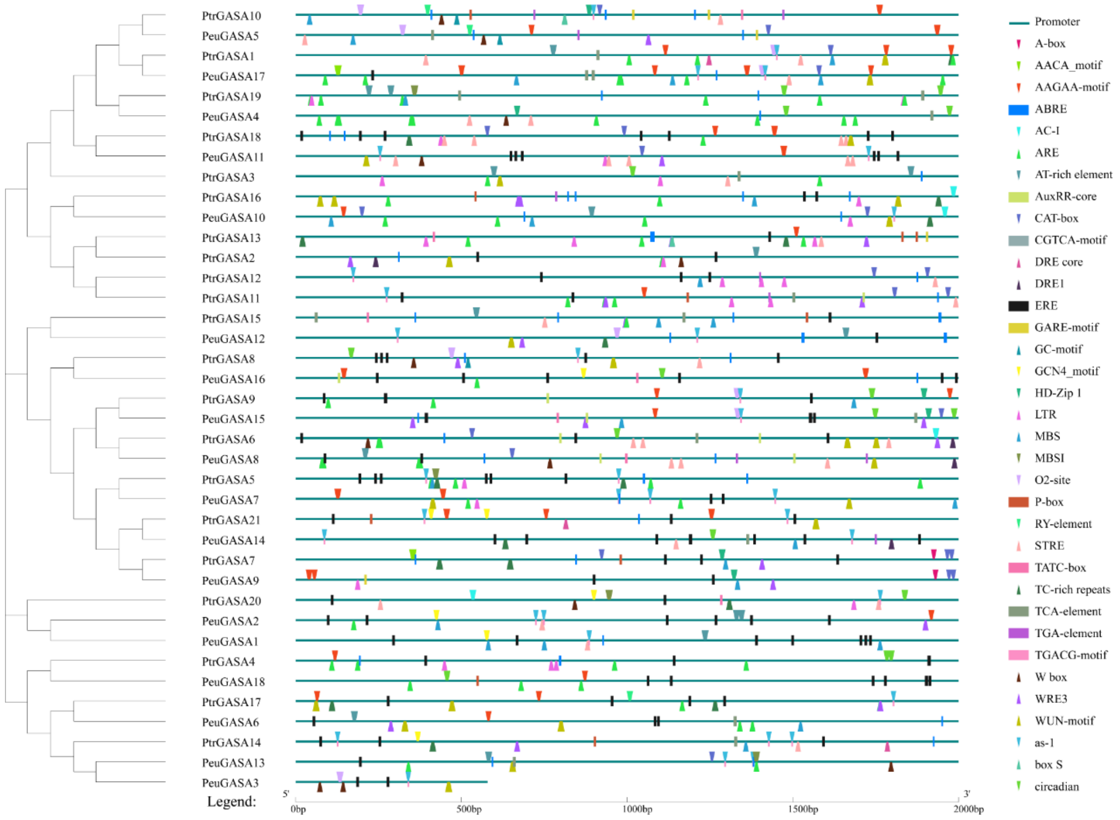

Figure S4. The position of the *cis*-acting elements on the promoter region of *GASAs*.

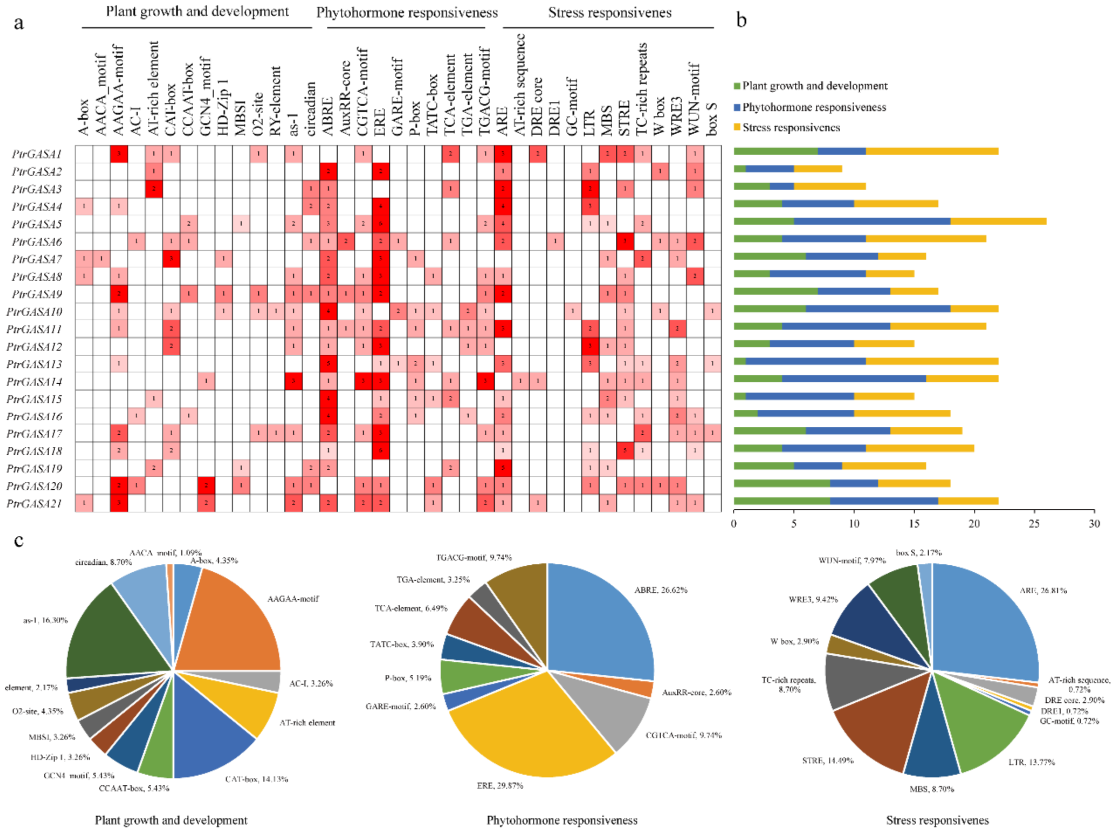

Figure S5. The *cis*-acting elements of *PtrGASA* genes. (**a**) Numbers and gradient red colors indicate the number of *cis*-acting elements; (**b**) Color-coded histograms indicate the number of *cis*-acting elements of genes in each category; (**c**) Pie charts show the proportion of different *cis*-acting elements in each category.

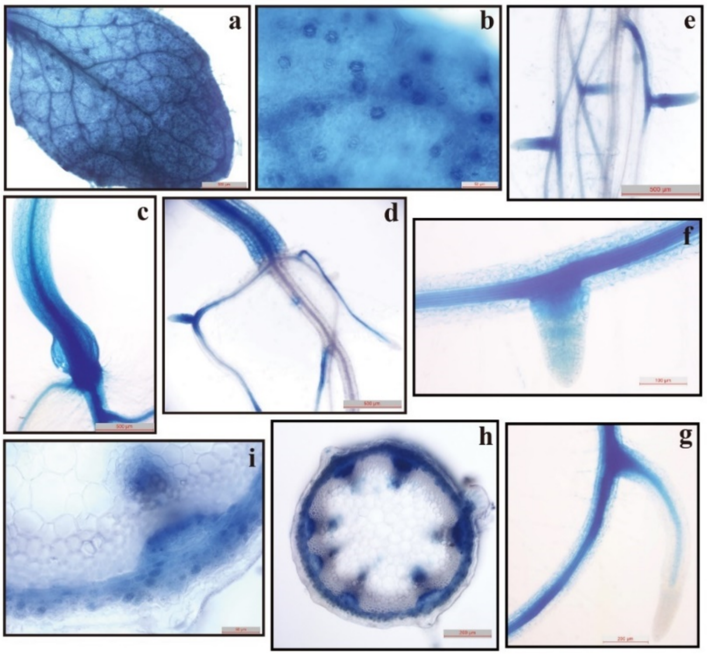

Figure S6. Transgenic *Arabidopsis* tissue under a microscope. (**a**) Leaf; (**b**) stoma; (**c**–**g**) hypocotyls and roots; (**h**,**i**) Stem crosscutting.

Table S1. The position of *GASA* gene family in *Populus.*

Gene	Gene Name	Position	
LOC105108948	*PeuGASA1*	NW_011500085.1 (253260..255106, complement)	
LOC105118698	*PeuGASA2*	NW_011501980.1 (1939..3437, complement)	
LOC105120240	*PeuGASA3*	NW_011505596.1 (579..1520)	
LOC105123460	*PeuGASA4*	NW_011499856.1 (2422403..2423496, complement)	
LOC105127637	*PeuGASA5*	NW_011499872.1 (1730061..1731124, complement)	
LOC105128593	*PeuGASA6*	NW_011499877.1 (999727..1000813,complement)	
LOC105128863	*PeuGASA7*	NW_011499878.1 (1475944..1477033, complement)	
LOC105128865	*PeuGASA8*	NW_011499878.1 (1472235..1473361, complement)	
LOC105128866	*PeuGASA9*	NW_011499878.1 (1469630..1470495, complement)	
LOC105128880	*PeuGASA10*	NW_011499846.1 (516539..517398)	
LOC105133442	*PeuGASA11*	NW_011499907.1 (13672..14941)	
LOC105135117	*PeuGASA12*	NW_011499921.1 (212676..214532, complement)	
LOC105139569	*PeuGASA13*	NW_011499976.1 (560472..561432, complement)	
LOC105140101	*PeuGASA14*	NW_011499986.1 (190386..191299)	
LOC105140102	*PeuGASA15*	NW_011499986.1 (204050..205174)	
LOC105140103	*PeuGASA16*	NW_011499986.1 (208383..209439)	
LOC105140300	*PeuGASA17*	NW_011499990.1 (62980..63949, complement)	
LOC105141680	*PeuGASA18*	NW_011500016.1 (91069..92453, complement)	
GWHGAAYU001570	*PeuGASA19*	GWHAAYU00000002 (26615279..26616111)	
**Gene Locus**	**Gene Name**	**Position**	**Homologous Gene**
Potri.001G254100	*PtrGASA1*	Chr01:26388610..26389799 reverse	*PeuGASA17*
Potri.001G297700	*PtrGASA2*	Chr01:30205607..30206690 forward	*PeuGASA19*
Potri.001G315500	*PtrGASA3*	Chr01:32117807..32119314 reverse	No
Potri.001G350600	*PtrGASA4*	Chr01:35731577..35732904 forward	*PeuGASA18*
Potri.002G022500	*PtrGASA5*	Chr02:1361599..1362424 forward	*PeuGASA7*
Potri.002G022600	*PtrGASA6*	Chr02:1364982..1366298 forward	*PeuGASA8*
Potri.002G022700	*PtrGASA7*	Chr02:1368371..1369242 forward	*PeuGASA9*
Potri.005G239000	*PtrGASA8*	Chr05:24568559..24569894 reverse	*PeuGASA16*
Potri.005G239100	*PtrGASA9*	Chr05:24571982..24573134 reverse	*PeuGASA15*
Potri.006G044400	*PtrGASA10*	Chr06:3162131..3163068 reverse	*PeuGASA5*
Potri.007G051300	*PtrGASA11*	Chr07:5014100..5015000 reverse	No
Potri.007G051400	*PtrGASA12*	Chr07:5029280..5029709 reverse	No
Potri.009G092600	*PtrGASA13*	Chr09:8437317..8438127 forward	No
Potri.012G076700	*PtrGASA14*	Chr12:10207178..10208272 forward	*PeuGASA3/13*
Potri.013G113400	*PtrGASA15*	Chr13:12673726..12675596 reverse	*PeuGASA12*
Potri.014G020100	*PtrGASA16*	Chr14:1815675..1816439 forward	*PeuGASA10*
Potri.015G071500	*PtrGASA17*	Chr15:9609662..9611128 forward	*PeuGASA6*
Potri.017G083000	*PtrGASA18*	Chr17:9992148..9993493 forward	*PeuGASA11*
Potri.017G124200	*PtrGASA19*	Chr17:13635455..13636699 reverse	*PeuGASA4*
Potri.019G083900	*PtrGASA20*	Chr19:11678588..11680344 forward	*PeuGASA1/2*
Potri.T155100	*PtrGASA21*	scaffold_621:5902..7033 reverse	*PeuGASA14*

Table S2. Physicochemical properties and subcellular localization prediction of GASAs in *Populus.*

Gene Name	Formula	Molecular Weight	Theoretical pI	Instability Index (II)	Aliphatic Index	GRAVY	Localization Predicted
PeuGASA1	$C_{521}H_{850}N_{152}O_{147}S_{14}$	12,184.32	9.47	54.87	71.91	−0.224	extr: 13, vacu: 1
PeuGASA2	$C_{521}H_{850}N_{152}O_{147}S_{14}$	12,184.32	9.47	54.87	71.91	−0.224	extr: 13, vacu: 1
PeuGASA3	$C_{494}H_{830}N_{156}O_{150}S_{17}$	11,900	8.88	40.03	75.96	−0.073	extr: 11, vacu: 3
PeuGASA4	$C_{461}H_{746}N_{130}O_{127}S_{14}$	10,590.63	9.2	40.4	71.89	−0.109	chlo: 6, extr: 5, cyto: 1, mito: 1, E.R.: 1
PeuGASA5	$C_{552}H_{890}N_{158}O_{159}S_{14}$	12,732.94	9.36	56.2	61.38	−0.397	extr: 9, vacu: 3, chlo: 1, golg: 1
PeuGASA6	$C_{518}H_{857}N_{159}O_{151}S_{17}$	12,273.5	8.88	30.55	80.09	0.134	extr: 8, vacu: 6
PeuGASA7	$C_{525}H_{884}N_{158}O_{161}S_{15}$	12,466.66	9.2	41.33	81.86	0.074	cyto: 4, extr: 3, nucl: 2, vacu: 2, chlo: 1, mito: 1, E.R.: 1
PeuGASA8	$C_{448}H_{735}N_{139}O_{134}S_{14}$	10,661.45	9.04	53.61	69.12	0.012	extr: 12, mito: 1, vacu: 1
PeuGASA9	$C_{440}H_{745}N_{141}O_{135}S_{13}$	10,587.4	9.16	43	84.16	0.023	extr: 12, chlo: 1, vacu: 1
PeuGASA10	$C_{417}H_{671}N_{113}O_{117}S_{15}$	9620.5	9.01	25.79	60.91	0.03	extr: 10, vacu: 2, golg: 2
PeuGASA11	$C_{521}H_{818}N_{152}O_{135}S_{15}$	11,852.06	9.59	43.65	55.19	−0.286	extr: 7, vacu: 5, chlo: 1, cyto: 1
PeuGASA12	$C_{417}H_{681}N_{127}O_{126}S_{17}$	10,034.79	8.6	53.7	63.3	−0.127	extr: 12, cyto: 1, vacu: 1
PeuGASA13	$C_{494}H_{830}N_{156}O_{150}S_{17}$	11,900	8.88	40.03	75.96	−0.073	extr: 11, vacu: 3
PeuGASA14	$C_{455}H_{766}N_{142}O_{137}S_{14}$	10,866.8	9.16	41.78	82.43	0.122	extr: 6, chlo: 3, vacu: 3, cyto: 1, E.R.: 1
PeuGASA15	$C_{444}H_{737}N_{135}O_{140}S_{15}$	10,687.4	8.61	59.73	73.82	0.092	extr: 12, vacu: 2
PeuGASA16	$C_{439}H_{714}N_{130}O_{129}S_{15}$	10,358.19	8	37.72	88.57	0.356	extr: 12, cyto: 1, vacu: 1
PeuGASA17	$C_{530}H_{857}N_{155}O_{149}S_{15}$	12,265.48	9.48	49.68	61.42	−0.262	extr: 9, vacu: 3, chlo: 1, golg: 1
PeuGASA18	$C_{1063}H_{1704}N_{280}O_{284}S_{19}$	23,560.07	9.76	81.44	52.16	−0.441	extr: 12, vacu: 1, golg: 1
PeuGASA19	$C_{421}H_{677}N_{119}O_{119}S_{15}$	9790.63	9.12	33.52	56.59	−0.205	chlo: 4, extr: 4,vacu: 3, cyto: 2, nucl: 1
PtrGASA1	$C_{554}H_{896}N_{162}O_{155}S_{15}$	12,787.1	9.54	41.77	64.27	−0.223	extr: 9, chlo: 3, vacu: 2
PtrGASA2	$C_{422}H_{679}N_{119}O_{119}S_{15}$	9804.66	9.12	37.23	56.59	−0.203	chlo: 4, extr: 3, vacu: 3, cyto: 2, nucl: 1, plas: 1
PtrGASA3	$C_{441}H_{703}N_{119}O_{136}S_{15}$	10,329.05	7.96	52.4	61.04	−0.014	extr: 13, vacu: 1
PtrGASA4	$C_{911}H_{1469}N_{247}O_{247}S_{19}$	20,443.33	9.73	71.58	49.95	−0.442	extr: 13, vacu: 1
PtrGASA5	$C_{518}H_{871}N_{157}O_{161}S_{15}$	12,355.47	9.11	44.96	76.95	−0.015	chlo: 6, nucl: 2, plas: 2, cyto: 1, mito: 1, vacu: 1, E.R.: 1
PtrGASA6	$C_{459}H_{756}N_{140}O_{136}S_{14}$	10,860.75	9.03	55.53	73.11	0.042	extr: 12, mito: 1,

							vacu: 1
PtrGASA7	$C_{440}H_{744}N_{142}O_{135}S_{13}$	10,600.4	9.16	48.37	83.17	0.026	extr: 13, vacu: 1
PtrGASA8	$C_{437}H_{706}N_{128}O_{131}S_{15}$	10,330.09	8.00	36.62	80.61	0.317	extr: 12, cyto: 1, vacu: 1
PtrGASA9	$C_{463}H_{754}N_{138}O_{139}S_{14}$	10,926.76	8.66	55.04	75.92	0.147	extr: 12, mito: 1, vacu: 1
PtrGASA10	$C_{551}H_{886}N_{158}O_{159}S_{14}$	12,716.9	9.36	57.86	58.02	−0.443	extr: 8, vacu: 4, golg: 2
PtrGASA11	$C_{493}H_{780}N_{130}O_{137}S_{14}$	11,169.25	9.05	47.3	67.94	−0.015	extr: 7, chlo: 4, vacu: 3
PtrGASA12	$C_{493}H_{780}N_{130}O_{137}S_{14}$	11,169.25	9.05	47.3	67.94	−0.015	extr: 7, chlo: 4, vacu: 3
PtrGASA13	$C_{429}H_{692}N_{120}O_{121}S_{15}$	9947.85	9.11	34.21	55.96	−0.245	extr: 9, cyto: 3, chlo: 1, E.R.: 1
PtrGASA14	$C_{496}H_{832}N_{154}O_{150}S_{18}$	11,930.08	8.75	36.72	77.71	−0.012	extr: 10, vacu: 3, chlo: 1
PtrGASA15	$C_{415}H_{689}N_{131}O_{124}S_{17}$	10,042.86	8.97	49.23	60.11	−0.221	extr: 12, cyto: 1, vacu: 1
PtrGASA16	$C_{417}H_{671}N_{113}O_{117}S_{16}$	9652.56	9.01	27.19	57.61	0.003	extr: 11, golg: 2, vacu: 1
PtrGASA17	$C_{490}H_{822}N_{154}O_{147}S_{17}$	11,767.88	8.88	35.07	79.45	0.081	extr: 11, vacu: 3
PtrGASA18	$C_{524}H_{822}N_{150}O_{137}S_{15}$	11,896.11	9.54	42.59	54.67	−0.305	extr: 8, vacu: 4, chlo: 1, cyto: 1
PtrGASA19	$C_{450}H_{727}N_{129}O_{125}S_{15}$	10,425.41	9.14	42.88	68.17	−0.13	chlo: 6, extr: 5, cyto: 1, mito: 1, E.R.: 1
PtrGASA20	$C_{521}H_{850}N_{162}O_{147}S_{14}$	12,184.32	9.47	54.87	71.91	−0.244	extr: 13, vacu: 1
PtrGASA21	$C_{463}H_{776}N_{142}O_{134}S_{14}$	10,924.97	9.36	45.95	82.43	0.131	extr: 6, chlo: 3, vacu: 3, cyto: 1, E.R.: 1

Table S3. Subcellular localization and prediction of potential cleavage sites of GASAs in *Populus.*

Gene Name	Len	cTP	mTP	SP	other	Loc	RC	TPle
PeuGASA1	110	0.037	0.014	0.973	0.025	S	1	20
PeuGASA2	110	0.037	0.014	0.973	0.025	S	1	20
PeuGASA3	109	0.075	0.03	0.793	0.073	S	2	21
PeuGASA4	95	0.035	0.028	0.971	0.005	S	1	24
PeuGASA5	116	0.014	0.03	0.946	0.015	S	1	25
PeuGASA6	113	0.01	0.038	0.946	0.044	S	1	21
PeuGASA7	118	0.005	0.082	0.375	0.678	_	4	-
PeuGASA8	102	0.111	0.003	0.937	0.008	S	1	24
PeuGASA9	101	0.084	0.004	0.948	0.012	S	1	23
PeuGASA10	88	0.011	0.015	0.995	0.024	S	1	25
PeuGASA11	106	0.092	0.035	0.501	0.017	S	2	24
PeuGASA12	91	0.015	0.004	0.982	0.052	S	1	28
PeuGASA13	109	0.075	0.03	0.793	0.073	S	2	21
PeuGASA14	103	0.039	0.006	0.96	0.017	S	1	23
PeuGASA15	102	0.076	0.003	0.968	0.015	S	1	24
PeuGASA16	98	0.042	0.011	0.95	0.022	S	1	22
PeuGASA17	113	0.02	0.066	0.844	0.014	S	2	24
PeuGASA18	222	0.017	0.017	0.723	0.114	S	2	20
PeuGASA19	88	0.09	0.051	0.948	0.017	S	1	25

PtrGASA1	117	0.031	0.025	0.903	0.018	S	1	24
PtrGASA2	88	0.073	0.045	0.957	0.02	S	1	25
PtrGASA3	96	0.018	0.017	0.987	0.021	S	1	27
PtrGASA4	191	0.017	0.018	0.763	0.097	S	2	20
PtrGASA5	118	0.011	0.059	0.293	0.64	_	4	-
PtrGASA6	103	0.056	0.002	0.963	0.017	S	1	24
PtrGASA7	101	0.073	0.005	0.956	0.014	S	1	23
PtrGASA8	98	0.052	0.011	0.95	0.019	S	1	22
PtrGASA9	103	0.032	0.003	0.979	0.018	S	1	24
PtrGASA10	116	0.014	0.028	0.95	0.015	S	1	25
PtrGASA11	102	0.055	0.098	0.946	0.003	S	1	25
PtrGASA12	102	0.055	0.098	0.946	0.003	S	1	25
PtrGASA13	89	0.041	0.057	0.972	0.013	S	1	26
PtrGASA14	109	0.035	0.021	0.913	0.063	S	1	21
PtrGASA15	91	0.11	0.005	0.855	0.049	S	2	28
PtrGASA16	88	0.011	0.016	0.995	0.025	S	1	25
PtrGASA17	109	0.025	0.017	0.94	0.044	S	1	21
PtrGASA18	107	0.075	0.022	0.751	0.013	S	2	24
PtrGASA19	97	0.04	0.024	0.971	0.008	S	1	29
PtrGASA20	110	0.037	0.014	0.973	0.025	S	1	25
PtrGASA21	103	0.044	0.005	0.97	0.015	S	1	23

Table S4. The gene-specific primers for qRT-PCR.

Gene	Forward Primer	Reverse Primer
PeuUBQ	5′-AGACCTACACCAAGCCCAAGAAGAT-3′	5′-CCAGCACCGCACTCAGCATTAG-3′
PeuGASA1/2	5′-TAGCAACGCTGCAAATTCTCC-3′	5′-AGGCTTGCATAACATGGGCA-3′
PeuGASA3/13	5′-ATGGCAGTACGTTCGCTTCTT-3′	5′-ACGCACTTGCATCTCACACA-3′
PeuGASA4	5′-ATTCCCTCTTGCTGGTCCTT-3′	5′-CCATAAGTCCCAGGAGGAACAC-3′
PeuGASA5	5′-ACATCACAGAGGCTCCAACC-3′	5′-TCCCATAAGTTCCAGCAGGC-3′
PeuGASA6	5′-CAGCTTGTTGTGAGAGGTGG-3′	5′-CCATGGGTGGTCATGTCAGTA-3′
PeuGASA7	5′-CGCTTCGCTTGTCGTATCTC-3′	5′-TATCTAGGTTGCCGGAAGTGC-3′
PeuGASA8	5′-GTGAACTCGAATCTTGCTGCG-3′	5′-ATGGGTAGTCAAGCTGGCG-3′
PeuGASA9	5′-TGTGCTCCATCTTGCCGAAG-3′	5′-CACGAGTAGTCAAGGTGGCA-3′
PeuGASA10	5′-CACGAGTGCCCTTGCTACAG-3′	5′-CGGTGGCAACTACCGGAAT-3′
PeuGASA11	5′-TTGCCATTTCCATGCTCCAA-3′	5′-TGTAGCAAGGGCACACAGC-3′
PeuGASA12	5′-GGTCAGTGCCAAGACAACATT-3′	5′-GTTGGCATAGCAAGGACAGGT-3′
PeuGASA14	5′-CATCTCTCCTTGTGCTCCGC-3′	5′-CGGGTAGTCATGGTGGCATAG-3′
PeuGASA15	5′-CTTCTTGTTCTCCAACTTGCCG-3′	5′-GGCAAGCGTCATAGTTACCG-3′
PeuGASA16	5′-GCTGTCCTCCTTGTAGGCAT-3′	5′-CCATAGCAGGGACACACGTC-3′
PeuGASA17	5′-ACAACGACAGGGCAACCAT-3′	5′-CAGTTGTTGTAGCAAGGGCA-3′
PeuGASA18	5′-GCTGTGTGTCTTATGGTGGT-3′	5′-AGGTGGTGTCTTAACTGGTGG-3′
PeuGASA19	5′-GTTGCTGTGTTCCCTCCTCTT-3′	5′-AAGGGCACTCGTGCTTGTTC-3′

Table S5. The gene-specific primers of *PeuGASA*s for PCR-amplified.

Gene Name	Forward Primer	Reverse Primer
PeuGASA4	5′-ATGGCTAGCCTCAGTCGCA-3′	5′-CAAGGGCACTTGGGTCTTCC-3′
PeuGASA5	5′-ATGTCGAGGAAGCCAAGCATTA-3′	5′-AAGGGCACTTGGGGCCTC-3′
PeuGASA8	5′-AAGGACACTTGCGTCTGCC-3′	5′-AAGGACACTTGCGTCTGCC-3′
PeuGASA10	5′-ATGAAGCCTGTTTTTGCAGCTATT-3′	5′-AAGGGCACTTCGGTTTGCC-3′
PeuGASA11	5′-ATGGCTAAGTTTGTTGCTGTCTTC-3′	5′-AAGGGCACTTGGGCCCTC-3′
PeuGASA15	5′-ATGGCTATGATCACCAAGTTGCT-3′	5′-AAGGACACTTGCGTCTGCC-3′
PeuGASA18	5′-ATGGCTTTCAAAGCTGTGTGTC-3′	5′-AAGGGCACTTGGGCTTGTTG-3′
PeuGASA17	5′-ATGGGCAAATCAAGCATTGCC-3′	5′-AAGGGCATTTGGGCCCTC-3′
PeuGASA15Pro	5′-GGATCCACGCGATTGTCTGGTTGGAAG-3′	5′-GAATTCCTGGGATGAACTAGGAAAGGGTC-3′
